# Genome-Wide Identification and Functional Characterization of the Dof Family in *Dendrobium officinale*

**DOI:** 10.3390/ijms26062671

**Published:** 2025-03-16

**Authors:** Shoujie Li, Weiping Zhang, Can Si, Jing Chen, Yuhan Huang, Muyi Li, Hanzhi Liang, Jun Duan, Chunmei He

**Affiliations:** 1 Key Laboratory of South China Agricultural Plant Molecular Analysis and Genetic Improvement, Guangdong Provincial Key Laboratory of Applied Botany, South China Botanical Garden, Chinese Academy of Sciences, Guangzhou 510650, China; sjli0801@163.com (S.L.); cans2013@163.com (C.S.); chenjing2022@scbg.ac.cn (J.C.); huangyuhan123@scbg.ac.cn (Y.H.); 18398712715@163.com (M.L.); lianghanzhi123@163.com (H.L.); duanj@scib.ac.cn (J.D.); 2 Key Laboratory of National Forestry and Grassland Administration on Plant Conservation and Utilization in Southern China, South China Botanical Garden, Chinese Academy of Sciences, Guangzhou 510650, China; wpzhang921@163.com; 3 University of Chinese Academy of Sciences, Beijing 100049, China

**Keywords:** *Dendrobium officinale*, Dof transcription factor, gene expression, yeast one-hybrid

## Abstract

The Dof gene family represents a class of plant-specific transcription factors that play crucial regulatory roles in various biological processes, including plant growth, development, and responses to abiotic stress. However, genome-wide identification and functional characterization of the Dof gene family remain unexplored in *Dendrobium officinale*. In this study, we performed a genome-wide identification and functional analysis of the DoDof gene family. A total of 28 Dof family members were identified and named *DoDof1–28* based on genome annotation data. Phylogenetic analysis classified these genes into four major groups (A–D) and further subdivided them into nine subfamilies. Gene structure analysis revealed that most *DoDofs* lack introns, with no distinct specificity observed among different subfamilies and considerable diversity within the same subfamily. Sequence alignment analysis demonstrated that all DoDof proteins contain a conserved Dof domain consisting of 52 amino acids, which includes a C2-C2 zinc finger motif and a DNA-binding domain. MEME analysis revealed that the conserved motif composition exhibits a certain degree of conservation among DoDof proteins, but significant differences exist across subfamilies. Expression pattern analysis demonstrated that *DoDofs* have exhibited diverse expression profiles across different developmental stages, tissues, and under abiotic stresses (such as low temperature, salinity, and drought) in *D. officinale*, suggesting their potential roles in plant development and stress responses. Subcellular localization analysis indicated that DoDof15, DoDof22, and DoDof24 are localized exclusively in the nucleus. Yeast one-hybrid assays revealed that DoDof22 binds to the promoter of the ABA receptor *DoPYL9*, while DoDof15 and DoDof24 bind to the promoter of the bHLH transcription factor *DobHLH68*. These results suggest that DoDof proteins may regulate the growth, development, and stress response processes of *D. officinale* by binding to the promoters of target genes. This study provides critical insights into the functional roles of Dof transcription factors in Orchidaceae family and establishes a theoretical foundation for molecular breeding and stress resistance improvement in *D. officinale*.

## 1. Introduction

Transcription factors (TFs) are nuclear-localized proteins that regulate various biological processes, including growth, development, and stress responses, by binding to specific DNA sequences and activating or repressing the expression of downstream target genes at the transcriptional level [1]. Among these TF families, the biological functions of many transcription factor families, such as WRKY, MYB, and NAC, have been extensively studied. The Dof (DNA-binding with one finger) family is a class of plant-specific transcription factors [2] that play crucial roles in plant development and stress responses [3,4,5]. In contrast to the WRKY and MYB superfamilies, the Dof family has a relatively limited number of members, typically not exceeding 50 in higher plants. For example, 36 and 30 members have been identified in *Arabidopsis thaliana* and *Oryza sativa*, respectively [6,7]. Notably, the Dof transcription factor has not been found in some lower plants, such as *Cyanidioschyzon merolae* and *Thalassiosira pseudonana* [8], suggesting that the Dof family may have been acquired during plant evolution and plays an important role in the transition from lower to higher plants.

The Dof proteins have a relatively small molecular weight and consist of four key regions: an N-terminal oligomerization site, a Dof domain, a nuclear localization signal, and a transcriptional activation domain [9]. Notably, the N-terminal region contains a zinc finger domain composed of 52 amino acid residues [10]. This domain contains four cysteine residues that coordinate a Zn^2+^ ion, forming a zinc finger loop [11]. These four cysteines are essential for the stability of the zinc finger loop and play a crucial role in binding metal ions and maintaining the conformation required for DNA binding. Mutations in any of these four cysteine residues can disrupt the zinc finger structure, preventing the protein from binding to the promoter DNA of target genes and thereby abolishing its regulatory activity [12]. The N-terminal zinc finger domain is highly conserved across species, from lower plants such as algae to higher plants like *A. thaliana* and *O. sativa*, indicating that this domain is crucial for the biological functions of Dof proteins. Studies have shown that the zinc finger domain of Dof specifically recognizes the core sequence 5′-(T/A)AAAG-3′ in the upstream regions of target genes [13]. Due to the short length of this sequence, many gene promoters contain this binding site [6]. However, not all genes containing this binding site are target genes of Dof transcription factors; most of these sites may be non-functional and cannot be recognized or bound by Dof proteins. The C-terminal region of Dof transcription factors has a transcriptional regulatory function. The amino acid sequence of the C-terminal transcriptional regulatory region is highly variable and lacks conservation, exhibiting functional diversity [14,15]. This region can act as either a transcriptional activator or repressor, modulating the activity of target gene promoters and thereby regulating transcriptional levels to fulfill its biological roles. For example, Dof1 (MNB1a) acts as a transcriptional activator through its C-terminal region, activating the expression of light-regulated genes and participating in light signal transduction and tissue-specific gene expression in *Zea mays*. In contrast, Dof2, despite its high sequence similarity with Dof1 in the Dof domain, acts as a transcriptional repressor due to differences in its C-terminal region [16].

Dof proteins are considered dual-functional transcription factors that play crucial regulatory roles in plant dormancy, germination, development, and various physiological processes. Dormancy and germination, which are strictly regulated by the coordinated expression of multiple genes, are complex processes in the plant life cycle [17,18]. Dof proteins regulate the expression of genes associated with plant dormancy and germination, which is of great significance for analyzing plant life processes [19,20]. For example, the RGL2-DOF6 complex activates the expression of *GATA12* to regulate seed dormancy in *A. thaliana* [21]. Dof proteins are also involved in the growth and development of plant tissues, such as root growth, hypocotyl elongation, and the development and senescence of leaves and floral organs [22,23,24,25]. For instance, *PbDof9.2* encodes a nuclear-localized protein that regulates the expression of the *FT* gene by suppressing the activity of the *PbTFL1a* and *PbTFL1b* promoters, thereby delaying the flowering time in *Pyrus bretschneideri* [26]. Notably, Dof transcription factors are involved in the response to abiotic stress in plants, enhancing their stress tolerance. For example, *ThDOF1.4* improves salt and osmotic stress tolerance by increasing proline levels and enhancing reactive oxygen species (ROS) scavenging capacity in *Tamarix hispida* [27]. The proteins encoded by *SlCDF1–5* are nuclear-localized and exhibit diverse transcriptional activation in *Solanum lycopersicum*. These genes are induced under osmotic, salt, heat, and low-temperature stress, and overexpression of *SlCDF1* or *SlCDF3* enhances drought and salt tolerance in *A. thaliana* [28]. These studies demonstrate that Dof proteins play a crucial role in plant stress responses and may provide valuable genetic resources and strategies for plant breeding. The research remains notably limited on the functions of Dof genes. Most Dof proteins have yet to be identified or characterized across various plant species, leading to an incomplete understanding of the regulatory networks and hindering their potential application in plant breeding and related fields.

The Orchidaceae family is one of the largest families of flowering plants. *D. officinale*, a member of the *Dendrobium* genus within the Orchidaceae family, is a perennial herbaceous plant and a valuable traditional medicinal material. This study identified and analyzed the Dof family and the composition of conserved motifs, as well as the expression patterns under different developmental stages, tissues, and stress treatments. Additionally, potential DoDof target genes were identified and analyzed, and the binding of DoDof proteins to these genes was examined using yeast one-hybrid analysis. A comprehensive study of the DoDof family and its target genes provides valuable insights into the functions, clarifies future research directions, and offers clues for the application of DoDof transcription factors in orchids.

## 2. Results

### 2.1. Identification and Classification of Dof Family in D. Officinale

**Figure 1:**
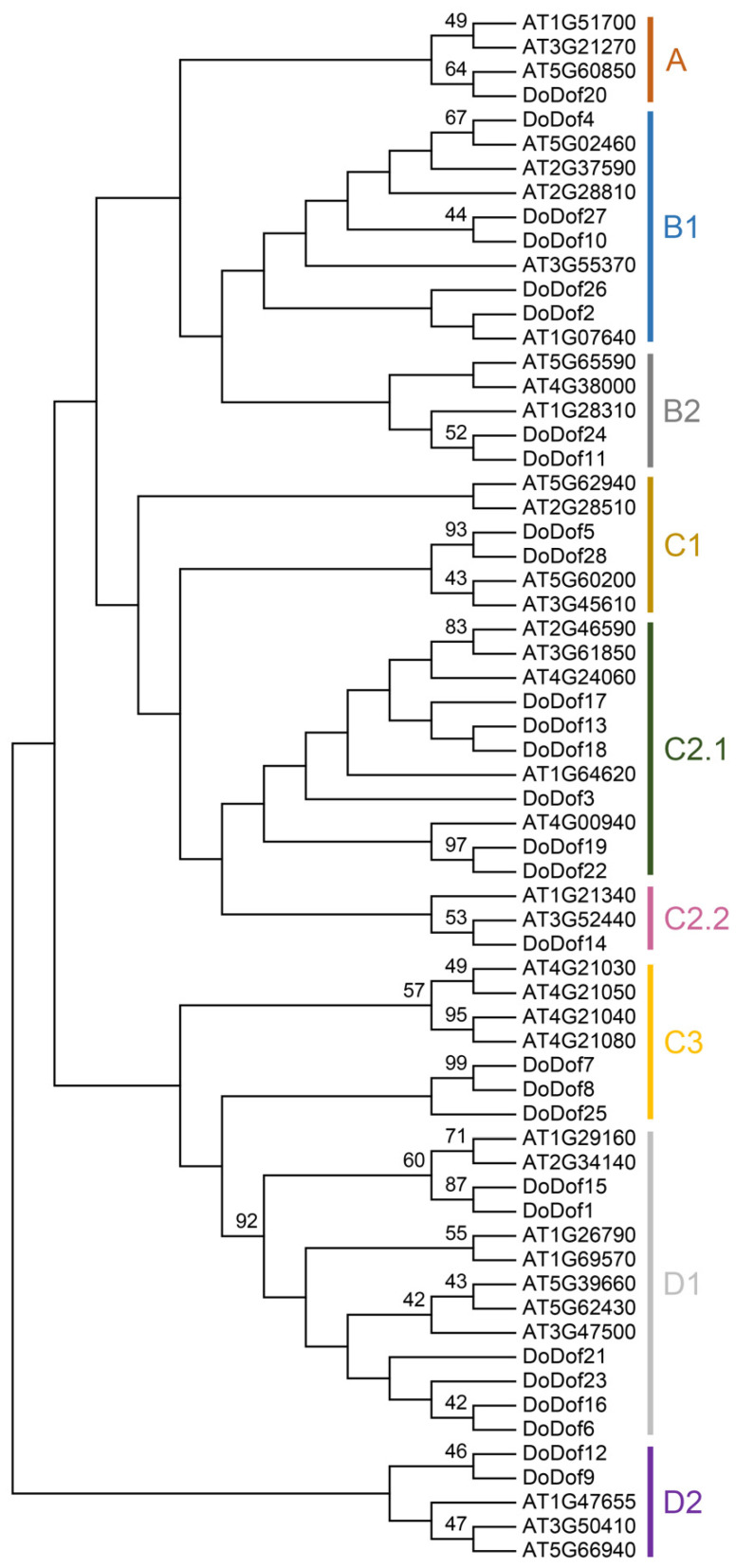
Phylogenetic tree of Dof proteins in *D. officinale* and *A. thaliana*. The phylogenetic tree was constructed using the neighbor-joining (NJ) method with 1000 bootstrap replications based on full-length protein sequences from 28 DoDofs and 36 AtDofs by MEGA 7.0.

**Figure 2:**
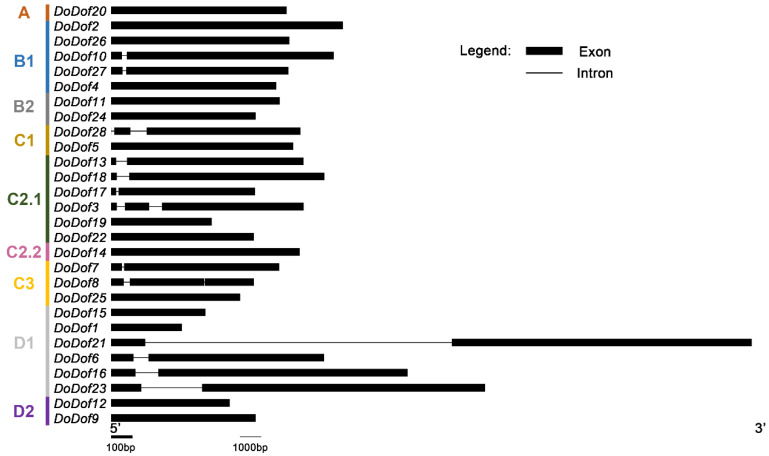
Gene structure analysis of *DoDof* genes in *D. officinale*. The intron and exon information of *DoDof* genes were obtained by whole genome sequencing GFF file of *D. officinale*. *DoDof* genes structure were mapped using GSDS 2.0 software.

A total of 28 Dof family members were identified and named *DoDof1–28* based on the annotation file of the *D. officinale* genome. These genes are mainly distributed across different scaffolds, with three pairs of genes located on the same scaffold. For example, *DoDof2* and *DoDof3* are located on scaffold716, and *DoDof24* and *DoDof25* are located on scaffold1013 (Table S1). The coding sequence (CDS) lengths of the 28 DoDof proteins range from 330 to 1551 bp, with 23 genes shorter than 1000 bp (Table S1). To further analyze the family classification, we conducted a phylogenetic analysis of the 28 DoDof proteins from *D. officinale* and the 36 AtDof proteins from *A. thaliana* using the neighbor-joining method (Table S2). These Dof proteins can be classified into four groups (A–D), which were further subdivided into nine subfamilies: A, B1, B2, C1, C2.1, C2.2, C3, D1, and D2. As with other plants, group A contains only one subfamily, while groups B, C, and D have at least two subfamilies. Group C has the most subfamilies, including C1, C2.1, C2.2, and C3 (Figure 1). Group A has the fewest members, only containing DoDof20, while Group C has the most members, with 12 members (Figure 1). Gene structure analysis revealed that the *DoDofs* exhibit significant structural diversity. For instance, within the C3 subfamily, one member contains one intron, another contains two introns, and yet another lacks an intron (Figure 2). Most *DoDofs* lack introns, with all members of subfamilies A, B2, C2.2, and D2 devoid of introns (Figure 2). Among all genes, 15 genes have no introns, 10 genes have one intron, and only three genes have two introns (Figure 2). These findings indicate that the gene structure of *DoDofs* is not conserved.

### 2.2. Sequence Alignment and Gene Structure Analysis of DoDof Proteins

**Figure 3:**
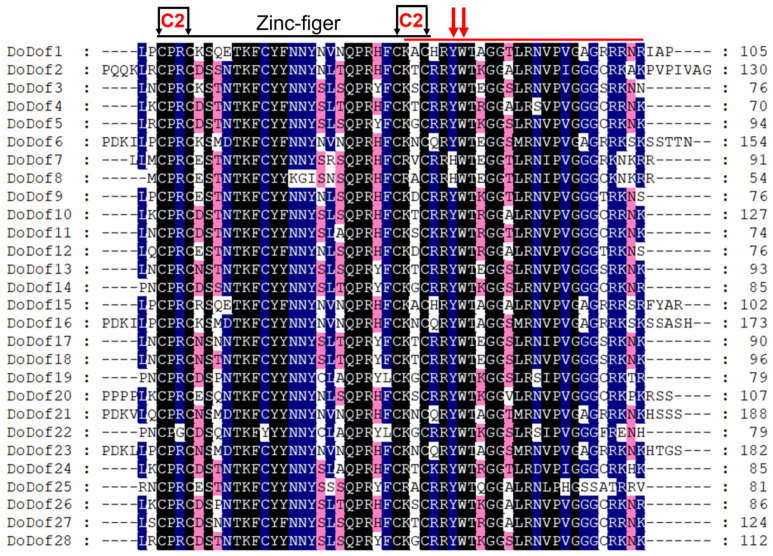
Multiple sequence alignment of DoDof proteins in *D. officinale*. Dof domain were aligned using ClustalX 2.1 and export the file in FASTA format, amino acid conserved residues were displayed using Genedoc 3.0. Different colors indicate different levels of conservation in residues, black, blue and pink indicate 100%, 75%, and 50% identical residues, respectively. Black arrows indicate cysteine residues that are likely contributing to the formation of a C2–C2 Zn finger. A red bar marks a region outside the Zn finger that plays a role in DNA binding. Red arrows highlight conserved aromatic amino acid residues that are essential for DNA interaction.

**Figure 4:**
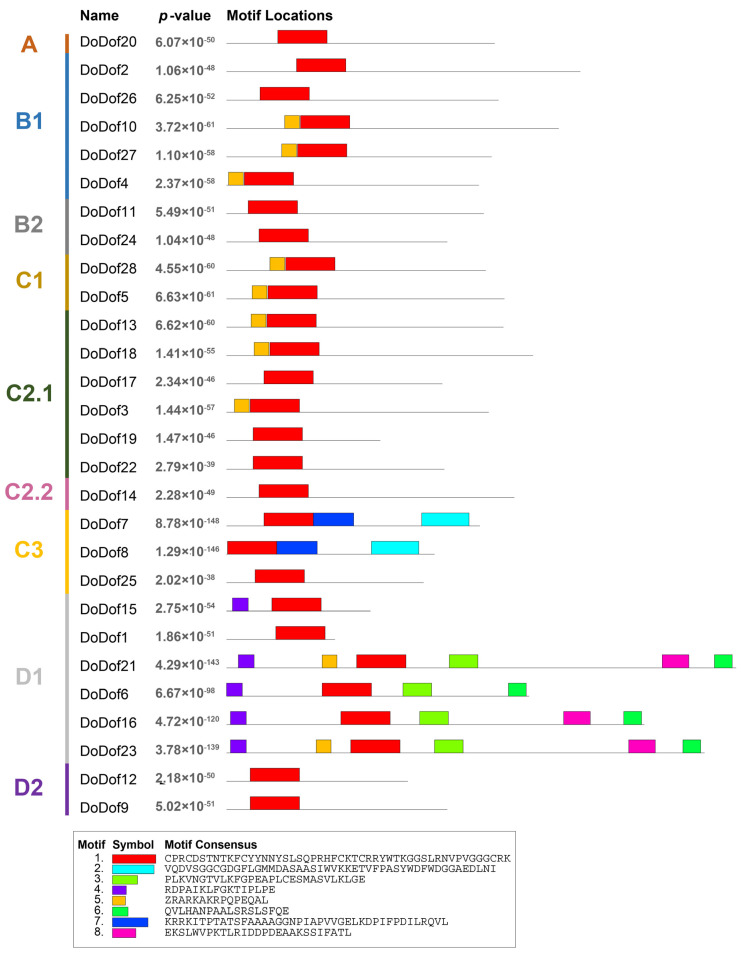
Conserved motif analysis of DoDof protein in *D. officinale*. DoDof protein sequence were imported into MEME online website for conserved domain analysis. The different subfamilies of the DoDof genes are shown in different colors on the left. The sequence of conserved motifs is presented at the bottom.

Sequence alignment analysis of DoDof proteins revealed that contain a conserved domain composed of 52 amino acids, which includes a conserved CX2CX21CX2C (C2–C2) motif and a DNA-binding domain (Figure 3). Within the C2–C2 zinc finger domain, there are four Cys residues that covalently bind to Zn^2+^ to form a single zinc finger C2–C2 domain, which plays a crucial role in DNA binding (Figure 3). The C2–C2 domain cannot function when Cys is replaced by His [12]. Adjacent to the zinc finger domain is a DNA-binding motif, which is present in most DoDof proteins and contains two conserved amino acids, Tyr (Y) and Trp (W). However, the Tyr (Y) is mutated to His (H) in DoDof7 and DoDof8 (Figure 3). The MEME online website was used to predict the conserved motifs and further analyze the motif characteristics of DoDof proteins. Interestingly, the motif composition of DoDof proteins shows a certain degree of conservation in that all DoDofs containing motif1 (Figure 4). Some motifs are unique to specific subfamilies, for example, motif2 and motif7 are only present in the C3 subfamily, while motif3, motif4, motif6, and motif8 are only found in the D1 subfamily. These motifs may play important roles in the functioning of genes, leading to the diversity of functions within the Dof gene family.

### 2.3. Expression Patterns of DoDof Genes in Different Developmental Stages and Tissues

**Figure 5:**
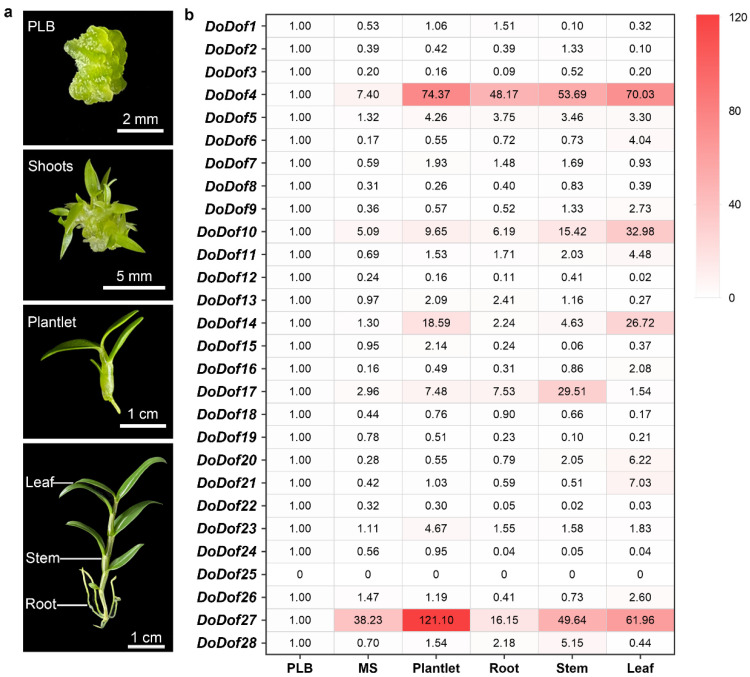
*DoDof* genes expression analysis of *D. officinale* in different development stages and tissues. (**a**) The three developmental stages of *D. officinale* are protocorm-like body (PLB), shoots (MS), and plantlet. The three organs of the adult plant (about 10 cm) are the root, stem, and leaf. (**b**) Heatmap of expression pattern of 28 *DoDof* genes by qRT-PCR. The values are the mean values of Ct for three technical repeats.

We analyzed the expression patterns of *DoDofs* in different developmental stages and tissues, including protocorm-like bodies (PLBs), shoots derived from PLB, plantlets, and the rhizomes, stems, and leaves of adult plants. The qPCR results revealed that, except for *DoDof25*, which was not detected, all other genes were expressed, with *DoDof4* and *DoDof27* having relatively high expression levels in plantlets (Figure 5). The expression levels of *DoDof3*, *DoDof8*, *DoDof12*, *DoDof18*, *DoDof19*, *DoDof22*, and *DoDof24* were highest in PLBs and significantly decreased as PLBs developed into shoots (Figure 5). The expression levels of *DoDof4*, *DoDof5*, *DoDof7*, *DoDof15*, *DoDof23*, and *DoDof27* were highest in plantlets and relatively low in PLBs and shoots, suggesting that these genes may play important roles in plantlets development (Figure 5). Notably, the expression levels of *DoDof6*, *DoDof9*, *DoDof10*, *DoDof11*, *DoDof14*, *DoDof16*, *DoDof20*, *DoDof21*, and *DoDof26* were highest in leaves, implying that these genes are involved in leaf development of *D. officinale* (Figure 5). The expression levels of *DoDof2*, *DoDof17*, and *DoDof28* were highest in stems and lowest in leaves (Figure 5). These results indicate that the expression patterns of *DoDofs* are diverse and they may play important roles in the organ development of *D. officinale*.

### 2.4. Expression Patterns of DoDof Genes Under Different Stress Treatments

**Figure 6:**
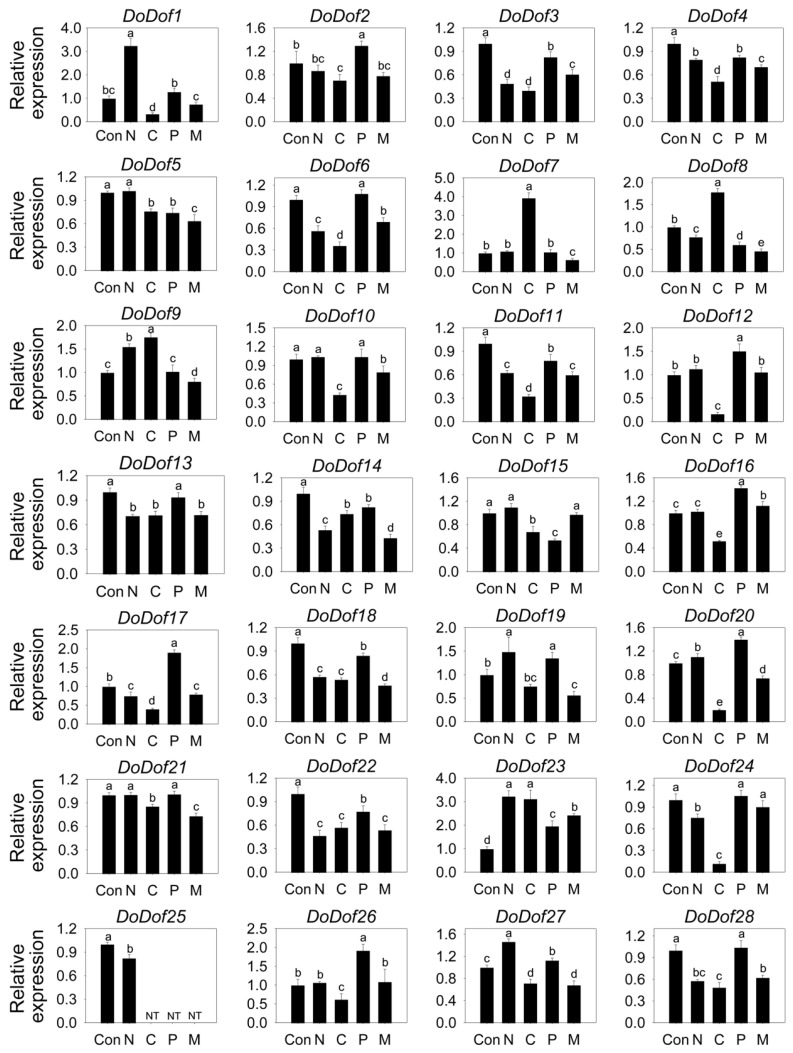
Expression pattern of *DoDof* genes in *D. officinale* under stress treatment. Seedlings untreated, 250 mM NaCl treatment, 4 °C treatment, 15% polyethylene glycol-6000 treatment and 300 mM Mannitol treatment are denoted by the letters Con, N, C, P, and M, respectively. Each data bar represents the mean ± standard deviation (SD) of three replicates. ND, not detected. Different lowercase letters indicate statistically significant differences (*p* < 0.05), while the same letters indicate no significant difference.

Dof transcription factors are widely involved in stress-response processes in plants. The expression patterns of *DoDof* genes were analyzed under salt, low temperature, 15% PEG-6000, and mannitol treatments. All 28 *DoDofs* showed altered expression under low-temperature stress, with the expression levels of 24 genes significantly decreased, while the expression of *DoDof7*, *DoDof8*, *DoDof9*, and *DoDof23* significantly increased (Figure 6). Under salt treatment, the expression of *DoDof1*, *DoDof9*, *DoDof23*, and *DoDof27* was significantly upregulated, while those of *DoDof3*, *DoDof4*, *DoDof6*, *DoDof8*, *DoDof11*, *DoDof13*, *DoDof14*, *DoDof17*, *DoDof18*, *DoDof22*, *DoDof24*, *DoDof25*, and *DoDof28* were significantly downregulated (Figure 6). Notably, the expression of *DoDof25* was significantly reduced under salt stress and was undetectable in all other treatments (Figure 6). Although both PEG (osmotic stress) and mannitol (dehydration stress) are used to simulate drought stress treatments, *DoDofs* exhibit distinct expression trends under these two treatments, with most of the genes showing opposite expression patterns. Compared to untreated plants, the expression of *DoDof2*, *DoDof6*, *DoDof10*, *DoDof12*, *DoDof13*, *DoDof16*, *DoDof17*, *DoDof19*, *DoDof20*, *DoDof21* and *DoDof28* were significantly upregulated or remained unchanged under PEG treatment, while these genes were significantly downregulated under mannitol treatment (Figure 6). It is noteworthy that *DoDof25* was significantly downregulated under salt treatment and undetected under low temperature, PEG and mannitol treatments (Figure 6). These results indicate that *DoDofs* play a significant role in the response to stress and may primarily act as negative regulators in *D. officinale*.

### 2.5. Subcellular Localization Analysis of DoDof15, DoDof22, and DoDof24

**Figure 7:**
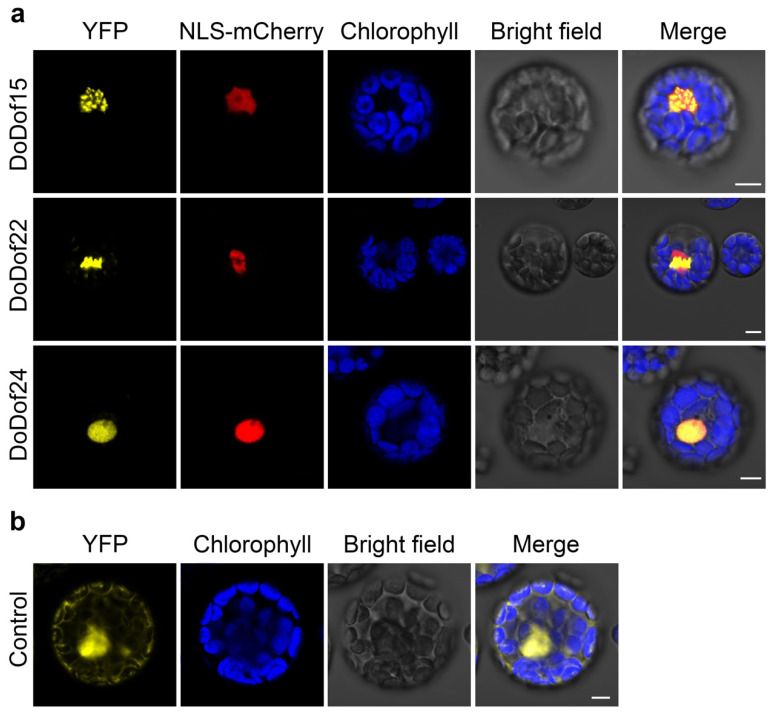
Subcellular localization of three DoDof proteins in *Arabidopsis* protoplasts. (**a**) Localization of DoDof15-YFP, DoDof22-YFP, and DoDof24-YFP fusion proteins with the nuclear marker NLS-mCherry. (**b**) The control YFP was co-transfected with empty YFP and NLS-mCherry plasmids. Scale bars = 5 μm.

Transcription factors perform their transcriptional regulatory roles within the nucleus [29]. Subcellular localization predictions of DoDof proteins also showed that these proteins are localized in the cell nucleus using WoLF PSORT (https://wolfpsort.hgc.jp/, accessed on 12 January 2025) online website (Table S3). To further analyze the localization of DoDof proteins, we selected representative genes from each subfamily based on their tissue-specific expression patterns: DoDof20 (Group A) was chosen due to its significant expression in leaves, suggesting a role in leaf-related processes; DoDof24 (Group B) and DoDof22 (Group C) were prioritized for their high expression in PLBs, indicating potential functions in PLB development; and DoDof15 (Group D) was selected for its prominent expression in plantlets, highlighting its importance in early growth regulation. These expression profiles provided functional insights to guide subsequent subcellular localization and functional analyses. However, DoDof20 could not be successfully cloned, so DoDof15, DoDof22, and DoDof24 were selected for further investigation. The DoDof-YFP transient expression vectors were constructed using yellow fluorescent protein (YFP) as the fluorescent marker. When these vectors were transiently expressed in protoplasts of *A. thaliana*, the yellow fluorescence of DoDof15-YFP, DoDof22-YFP, and DoDof24-YFP primarily appeared in the nuclear region and overlapped with the red fluorescence of the nuclear localization marker NLS-mCherry, indicating that DoDof15, DoDof22, and DoDof24 are localized in the nucleus. (Figure 7a). As a positive control, the yellow fluorescence of 35S: YFP was observed in the cell membrane, cytoplasm, and nucleus, showing a lack of specific localization (Figure 7b).

### 2.6. Binding Analysis of Candidate Target Genes for DoDof15, DoDof22, and DoDof24

**Figure 8:**
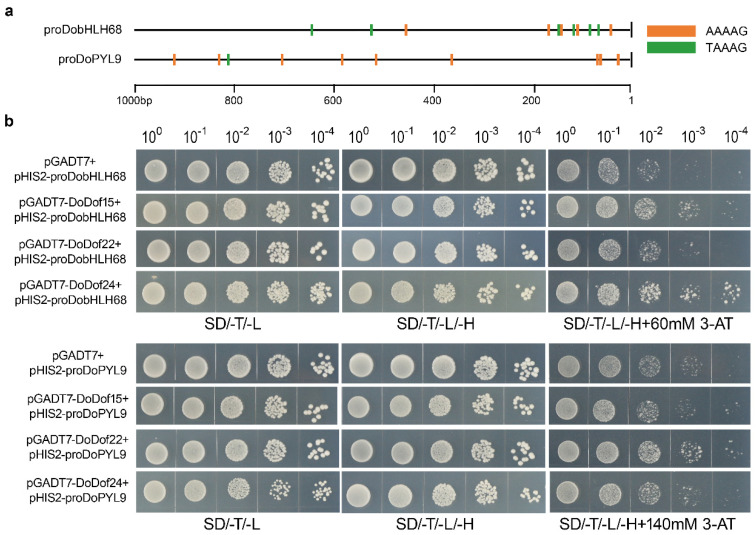
Validation of three DoDof proteins binding core sequence 5′-(T/A)AAAG-3′. (**a**) The number of core elements contained in the *DobHLH68* and *DoPYL9* promoters 1000 bp. (**b**) Yeast one-hybrid verification of DoDof15, DoDof22, and DoDof24 binding to *DobHLH68* and *DoPYL9* promoters, respectively. SD/-T/-L: a yeast culture medium without tryptophan and leucine; SD/-T/-L/-H: a yeast culture medium lacking tryptophan, leucine, and histidine; SD/-T/-L/-H + 3-AT: a yeast culture medium excluding tryptophan, leucine, and histidine, supplemented with 3-AT at specific concentrations (60 mmol/L for DobHLH68 and 140 mmol/L for DoPYL9).

Dof transcription factors bind to the core sequence 5′-(T/A)AAAG-3′ in the promoters of target genes, thereby regulating gene expression and achieving biological functions such as regulating plant growth and development, and responding to abiotic stress [30,31,32]. To date, the target genes regulated by DoDof transcription factors remain unidentified. The 1000 bp upstream sequences of the start codons of all genes were obtained through whole-genome data in *D. officinale*. Using the core sequence 5′-(T/A)AAAG-3′ as a probe, all promoters were scanned to identify genes containing the core sequence 5′-(T/A)AAAG-3′ in their promoter regions. A total of 27,164 genes were found to have at least one 5′-(T/A)AAAG-3′ core sequence in their promoter regions. Among these, 23,491 genes contain two or more 5′-(T/A)AAAG-3′ core sequences, and 18,734 genes have at least three 5′-(T/A)AAAG-3′ core sequences in their promoter sequences (Table S4). To improve the accuracy of identifying candidate target genes, only those containing at least three 5′-(T/A)AAAG-3′ core sequences were selected for further analysis. The bHLH transcription factor family exists in all eukaryotes and is involved in key processes of plant growth, development, and interaction with the environment [33]. Among these candidate target genes, the promoter of *DobHLH68* (Dca022885) contains eleven 5′-(T/A)AAAG-3′ core sequences (Figure 8a). Abscisic acid (ABA) is one of the most important plant hormones regulating stress responses, primarily exerting its precise regulatory effects through the ABA signaling pathway [33,34]. The ABA receptor protein PYL is a critical component of the ABA signaling pathway [35] and plays a significant role in stress responses [36,37,38]. Among the candidate target genes identified, the promoter region of the ABA receptor protein gene *DoPYL9* (Dca025790) contains ten 5′-(T/A)AAAG-3′ core sequences (Figure 8a). We analyzed the binding of DoDof15, DoDof22, and DoDof24 to the promoters of *DobHLH68* and *DoPYL9* in a yeast system, respectively. The negative control pGADT7 + proDobHLH68 and other combinations could grow normally in SD/-T/-L and SD/-T/-L/-H media. The growth of pGADT7 + proDobHLH68 was significantly inhibited, and even when DoDof22 was present, the growth of yeast was still inhibited in media containing 60 mM 3-amino-1, 2, 4-triazole (3-AT). However, yeast growth was better when DoDof15 and DoDof24 were present (Figure 8b and Figure S1). These results suggest that DoDof15 and DoDof24 can bind to the promoter of *DobHLH68* (proDobHLH68). The growth of pGADT7 + proDoPYL9 was significantly inhibited in media containing 140 mM 3-AT. In the presence of DoDof22, yeast growth was significantly improved, while no significant change was observed in the presence of DoDof15 and DoDof24 (Figure 8b and Figure S1), which suggests that DoDof22 binds to the promoter of *DoPYL9* (proDoPYL9).

## 3. Discussion

### 3.1. Dof Transcription Factors Are Ubiquitous in Plants and Possess a Conserved Zinc Finger Domain

The first Dof protein, ZmDof1, was identified in maize, and its binding domain includes a C2–C2 zinc finger motif [39]. Since this discovery, an increasing number of Dof family members have been identified across diverse plant species, ranging from single-celled algae, and mosses to flowering plants, such as *A. thaliana* [7], *Brassica rapa* L. ssp. *pekinensis* [40], *Cucumis sativus* [41], and *Betula platyphylla* [42]. Notably, only one Dof transcription factor has been identified in *Chlamydomonas reinhardtii*, *Volvox carteri*, and *Micromonas pusilla* [8,43]. In contrast, more complex plant species exhibit greater diversity, with the moss *Physcomitrella patens* containing 19 *Dof* genes and the gymnosperm *Pinus taeda* harboring 10 *Dof* genes [8]. Extensive studies have identified and characterized numerous Dof genes in angiosperms [9]. In the case of *D. officinale*, a member of the orchid family, 28 *DoDof* genes have been characterized. These findings suggest that Dof transcription factors are widely present in green plants and may play roles in functions specific to these plants. The N-terminus of these Dof proteins contains a conserved zinc finger domain, which has four cysteines. These four cysteines bind to a Zn^2+^ to form a zinc finger structure, which is necessary for the specific recognition of the core sequence 5′-(T/A)AAAG-3′ upstream of target genes. Among the 28 *DoDof* genes, the N-terminus of their protein sequences all contain a zinc finger domain (Figure 3). The Dof domain is not only a DNA-binding domain but also exhibits protein-protein interaction activity. Dof proteins can interact with other transcription factors, such as MYB, bZIP, and WRKY [9]. For example, the Dof protein SAD interacts with the R2R3MYB protein (GAMYB) to activate the transcription of endosperm-specific genes in *Hordeum vulgare* cv. Bomi [44]. These results indicate that the zinc finger domain is conserved in Dof transcription factors and plays a key role in the biological functions of this gene family.

### 3.2. Dof Transcription Factors Regulate Leaf Development in Plant

Leaves are one of the important organs in plants and serve as the primary sites for photosynthesis [45,46]. Stomata are distributed on the underside of leaves, regulating the exchange of gases and water vapor [47,48]. Dof transcription factors play a crucial role in regulating stomatal development. *SCAP1* encodes a Dof transcription factor, and the *scap1* mutant exhibits irregular guard cells, lacking the ability to control stomatal aperture, including CO_2_-induced stomatal closure and light-induced stomatal opening. For example, SCAP1 is a key regulator directly regulating stomatal function in *A. thaliana* [49]. The promoters of *AtDof2.4* and *AtDof5.8* exhibit specific activity in leaf primordia [50], suggesting their involvement in leaf initiation in *A. thaliana*. The activation tagging mutant *Dof5.1-D* exhibits an upward-curling leaf phenotype, while overexpression of the DNA-binding domain of *Dof5.1* results in a downward-curling leaf phenotype, indicating that *Dof5.1* regulates leaf axial patterning in *A. thaliana* [51]. Furthermore, *Dof5.8* encodes a Dof transcription factor, and its overexpression suppresses the formation of higher-order veins in cotyledons and leaves in *A. thaliana* [52]. Studies have demonstrated that Dof transcription factors are not only involved in leaf development but also play a role in leaf senescence. For instance, the Dof transcription factor CDF4 promotes leaf senescence by upregulating the transcription of ABA biosynthesis genes *nced2,3*, thereby increasing endogenous ABA levels. Additionally, CDF4 represses the expression of the *CAT2* gene, inhibiting the clearance of H_2_O_2_ and further accelerating leaf senescence [53]. *Dof2.1* is a JA-induced gene that is closely related to leaf senescence. Overexpression of *Dof2.1* promote leaf senescence in *A. thaliana*. Mechanistically, *Dof2.1* activates the *MYC2* promoter and enhances *MYC2* expression to drive leaf senescence [54]. Notably, the high expression levels of *DoDof6*, *DoDof9*, *DoDof10*, *DoDof11*, *DoDof14*, *DoDof16*, *DoDof20*, *DoDof21*, and *DoDof26* in the leaves suggest that these genes may be involved in leaf development or senescence and play significant roles in the regulation of leaf function in *D. officinale*.

### 3.3. Dof Transcription Factors Respond to Abiotic Stress in Plants

Dof transcription factors not only regulate in plant development but also play pivotal roles in plant stress responses to adverse conditions. Extensive research has demonstrated that Dof transcription factors can be induced by various abiotic stresses such as salt, drought, and low temperatures. For instance, most of *StDof* genes are upregulated under drought and salinity conditions in potatoes [14]. Two cold-induced genes, *OsDof1* and *OsDof9*, have been identified in *O. sativa* L., with *OsDof1* contributing to enhanced cold stress tolerance [55]. The Dof gene *CDF3* plays a significant role of abiotic stress responses in *A. thaliana*. It is strongly induced by drought, extreme temperatures, and abscisic acid (ABA) treatment. Mutants of *CDF3* exhibit heightened sensitivity to drought and low-temperature stress, whereas its overexpression improves tolerance to drought, cold, and osmotic stress in transgenic plants [56]. Research has revealed that Dof transcription factors are involved in stress response signaling pathways, such as ABA and ROS pathways, thereby enhancing plant stress tolerance. These transcription factors promote the accumulation of ABA by activating the expression of ABA biosynthesis genes and suppressing the expression of ABA catabolism genes. For example, the Dof transcription factor *CDF4* increases endogenous ABA levels by upregulating the transcription of the ABA biosynthesis gene *nced2,3* [53]. Similarly, *DAG1* directly represses the expression of the ABA catabolism gene *CYP707A2*, effectively increasing ABA content [57]. These findings suggest that Dof transcription factors likely promote the accumulation of ABA, activating the ABA signaling pathway and enabling plants to adapt to adverse environments such as drought, high salinity, and low temperatures. Under stress conditions, a large amount of ROS is accumulated in plants. While excessive ROS can damage cellular components, they also serve as vital signaling molecules [58]. Dof transcription factors have two functions in regulating ROS: promoting ROS accumulation and clearing ROS. For example, Dof transcription factors inhibit the expression of the *CAT2* gene, thereby inhibiting the clearance of H_2_O_2_ [53]. ROS plays a crucial role in the perception of abiotic and biotic stresses in plants, integrating different environmental signals, and activating stress response networks, which helps to establish defense mechanisms and stress resistance in plants [58]. The Dof transcription factor RsCDF3 directly binds to the promoters of the key plasma membrane-bound enzymes *RsRbohA* and *RsRbohC* that produce ROS in *Raphanus sativus* L., inhibiting their transcription, maintaining the homeostasis of ROS in cells, and thereby enhancing cold resistance [59]. In this study, the expression levels of all 28 *DoDof* genes were altered under low-temperature treatment, with four genes significantly upregulated and the rest downregulated. The expression levels of *DoDof1*, *DoDof9*, *DoDof23*, and *DoDof27* were significantly increased under salt stress. Similarly, some *DoDofs* were induced by PEG-6000 and mannitol (Figure 6). These studies suggest that Dof transcription factors may be involved in the stress response of *D. officinale*, indicating a conserved function in abiotic stress responses across plant species.

### 3.4. Dof Transcription Factors Show Diversity in Recognizing and Binding to Core Sequences

The single zinc finger domain of Dof transcription factors can specifically recognize and bind to the 5′-(T/A)AAAG-3′ sequence in the promoters of target genes, thereby regulating their expression and fulfilling the functions of Dof transcription factors. For instance, AtDof5.1 recognizes and binds to the TAAAGT motif, thus regulating the transcription of target genes in *A. thaliana* [51]. Dof transcription factors generally have a high affinity for the AAAG promoter sequence, and binding is weakened or prevented when AAAG is mutated [60,61]. However, this does not imply that all Dof transcription factors can bind to the 5′-(T/A)AAAG-3′ sequence; the sequences flanking this binding site may influence the binding function [10]. Although the promoters of *DobHLH68* and *DoPYL9* both contain the 5′-(T/A)AAAG-3′ sequence, the sequences at the ends of this binding site are not identical, which may contribute to the binding specificity of Dof transcription factors. Additionally, the C-terminal region of Dof proteins is crucial for protein-protein interactions and exhibits specificity. Dof transcription factors are likely to interact with other transcription factors to achieve the necessary promoter specificity. For example, the Dof transcription factor BPBF interacts with the MYB transcription factor GAMYB to activate the promoter of target genes, regulating of seed development and germination [62]. In this study, we found that the promoters of *DobHLH68* and *DoPYL9* both contain abundant 5′-(T/A)AAAG-3′ sequences. However, among the three selected DoDof genes, only DoDof15 and DoDof24 bound to the promoter of *DobHLH68* in yeast, while DoDof22 bound to the promoter of *DoPYL9* (Figure 8b). These research results indicate that the binding of Dof proteins to the 5′-(T/A)AAAG-3′ motif may be affected by the flanking sequences the motif and interacting proteins, and their regulatory roles may be a complex and diverse process on target genes. This indicates that more in-depth research is needed to clarify the biological functions of Dof in plant development and stress responses.

## 4. Materials and Methods

### 4.1. Plant Materials and Growth Conditions

The *D. officinale* plants used in this study were cultivated at the South China Botanical Garden, Chinese Academy of Sciences, Guangzhou, China. Additionally, tissue-cultured *D. officinale* seedlings are preserved in the laboratory. The experimental materials included protocorm-like bodies (PLBs), shoots (MS), plantlets (approximately 1.5 cm tall), and adult plants (about 10 cm tall), all of which were obtained under controlled tissue culture conditions. The culture room was maintained at 25 °C with a 12-h light/12-h dark photoperiod and 50 µmol m^−^^2^ s^−1^ light intensity. The basic culture medium was 1/2 MS medium [63], supplemented with 0.1% activated charcoal, 2% sucrose, and 0.6% agar (pH 5.4). To investigate stress responses, the plantlets (about 1 cm tall) were subjected to various abiotic stress treatments according to our previously established protocols [64]. The treatment conditions were as follows: 250 mM NaCl (Guangzhou Chemical Reagent Factory, Guangzhou, China) to simulate salt stress; 15% polyethylene glycol-6000 (PEG-6000) (Solarbio Science & Technology Co., Ltd., Beijing, China) to simulate osmotic stress; 300 mM mannitol (Macklin Biochemical Technology Co., Ltd., Shanghai, China) to simulate drought stress; and 4 °C low-temperature stress. Each stress treatment lasted 6 h, while the control group seedlings were cultured under normal conditions. Experiments utilized three independent biological replicates for PLBs, shoots, plantlets, and adult tissues, with five samples per condition analyzed in triplicate technical measurements, while stress-treated plantlets employed six biological replicates per treatment across three independent experiments, each with three technical replicates. Samples were collected and immediately frozen in liquid nitrogen for 15 min after treatment, followed by RNA extraction for subsequent analysis. The PLBs, MS, plantlets, and roots, stems, and leaves from adult *D. officinale* plants were processed similarly for qRT-PCR analysis.

For subcellular localization experiments, an appropriate quantity of Columbia-type *A. thaliana* seeds was placed in centrifuge tubes with distilled water and incubated in the dark at 4 °C for 2–3 days. Subsequently, the seeds were sown on a substrate mixture of nutrient soil and perlite (2:1, *v*/*v*) and transferred to a growth chamber maintained at 22 ± 2 °C with a 16-h light/8-h dark photoperiod, light intensity of 80 μmol/m^2^/s, and relative humidity of 80%. When the Arabidopsis reached 4–6 weeks of age, protoplasts were isolated from leaves using previously described methods [65].

### 4.2. Identification of DoDof Transcription Factors and Construction of Phylogenetic Tree

The genomic data of *D. officinale* were downloaded from the National Center for Biotechnology Information (NCBI) Genomic Database (https://www.ncbi.nlm.nih.gov/ (accessed on 1 January 2025)) under the project PRJNA262478 [66]. The *D. officinale* genome database was searched using the keyword “Dof” to identify genes containing this term in their annotations. BlastX alignment was performed on NCBI, along with phylogenetic analysis, to remove duplicate or incorrect DoDof sequences. Ultimately, 28 Dof proteins were identified from the *D. officinale* genome. The amino acid sequences of the DoDof proteins were then submitted to ProtParam (https://web.expasy.org/protparam/ (accessed on 1 January 2025.)) [67] to calculate their physicochemical properties and to WoLF PSORT (https://wolfpsort.hgc.jp/ (accessed on 1 January 2025)) for subcellular localization prediction.

Multiple sequence alignment of the full-length amino acid sequences of DoDof proteins was performed using Clustal X 2.0 software [68]. Based on the alignment results, a phylogenetic tree was constructed using the neighbor-joining (NJ) method [69] in MEGA 7 software [70]. The authenticity of the tree was tested by performing 1000 bootstrap replications, resulting in the construction of a phylogenetic tree of Dof proteins from *D. officinale* and *A. thaliana* (Table S2).

### 4.3. Bioinformatics Analysis of DoDof Proteins

Based on the FASTA alignment file obtained from Clustal X 2.0 software, multiple sequence alignment of the full-length amino acid sequences of DoDof proteins was performed using GENEDOC 3.0 software [71] with default parameters. The phylogenetic tree of the DoDof family proteins was constructed using the neighbor-joining method based on the multiple sequence alignment, and a Newick Tree file was exported. The gene annotation information of the DoDof family members was extracted from the GFF3/GTF file of *D. officinale* and submitted to the GSDS2.0 online website (https://gsds.gao-lab.org/ (accessed on 6 January 2025)) for gene structure visualization [72]. The DoDof proteins amino acid sequences were submitted to the MEME online website (https://meme-suite.org/meme/tools/meme (accessed on 6 January 2025)) for conserved motif analysis, with the number of conserved motifs set to 8 and other parameters maintained at default values. The conserved motif information was subsequently visualized [73].

### 4.4. RNA Extraction, cDNA Synthesis, and qRT-PCR Analysis

Total RNA was extracted from PLBs, shoots, plantlets, roots/stems/leaves of adult plants, and stress-treated plantlets using the HiPure HP Plant RNA Mini Kit R4165-02 (Magen Biotechnology Co., Ltd., Guangzhou, China). RNA purity and concentration were determined using the Nanodrop 2000C spectrophotometer (Thermo Scientific, Wilmington, DE, USA). First-strand cDNA synthesis was performed using the All-in-One First-Strand Synthesis MasterMix MD80101 (Magen Biotechnology Co., Ltd., Guangzhou, China), and the cDNA was quantified and stored at −20 °C for subsequent analysis. The qRT-PCR primers were designed (Table S5) and synthesized by Sangon Biotech Co., Ltd. (Shanghai, China). Using ACTIN (NCBI accession number: JX294908) as the reference gene [74], the relative expression levels of *DoDof* genes were determined across different developmental stages, tissues, and stress treatment samples in *D. officinale*. The qRT-PCR reactions were performed according to the protocol of Magen Universal SYBR qPCR Mix MD70101 (Magen Biotechnology Co., Ltd., Guangzhou, China) on the Roche LightCycler 480 system (Roche, Basel, Switzerland). The thermal cycling conditions consisted of an initial denaturation at 95 °C for 60 s, followed by 40 cycles of 95 °C for 10 s and 60 °C for 10 s. The gene relative expression levels were calculated using the 2^−ΔΔCt^ method [75] to determine the relative differences in gene expression between samples. The experimental data were presented as the average of three biological replicates, and graphs were created using Sigmaplot 15.0 software (Systat Software Inc., San Jose, CA, USA), followed by significance analysis.

### 4.5. Subcellular Localization Analysis

To investigate the subcellular localization of DoDof proteins, primers (Table S6) were designed to amplify the full-length coding sequences of *DoDof15*, *DoDof22*, and *DoDof24* genes. The pSAT6-EYFP-N1 vector [76] was digested with EcoRI, and the digested product was purified. The PCR-amplified target genes were ligated into the linearized vector using homologous recombination. The recombinant plasmids were transformed into *Escherichia coli* DH5α competent cells (Weidi Biotechnology Co., Shanghai, China). After transformation, single colonies were selected for colony PCR screening, and positive clones were subsequently sequenced. The correctly sequenced strains were cultured in a liquid medium for amplification. The recombinant plasmids (DoDof15-YFP, DoDof22-YFP, and DoDof24-YFP) were extracted and co-transformed with the nuclear localization marker NLS-mCherry into mesophyll protoplasts using polyethylene glycol (PEG)-mediated transformation [65]. The transformed protoplasts were incubated in darkness for 8–10 h. Fluorescence expression was observed using a Leica TCS SP8 STED 3× super-resolution confocal microscope (Leica Camera AG., Solms, Germany). The excitation and emission wavelengths were 514 nm and 527 nm for YFP, and 587 nm and 610 nm for the nuclear marker mCherry, respectively.

### 4.6. Analysis of the Core Sequence 5′-(T/A)AAAG-3′ in the Promoter of D. Officinale

To evaluate the distribution characteristics and potential regulatory roles of Dof recognition sequences in the genome of *D. officinale*, the upstream 1000 bp sequences of all genes were extracted as promoter regions using Tbtools software, version 2.121 [77]. Subsequently, the Dof recognition sequences 5′-(T/A)AAAG-3′ was identified within the extracted sequences using regular expression matching, specifically targeting sequences beginning with either T or A followed by AAAG. The occurrence frequency of this core sequences in the promoters of each gene was counted, and its distribution pattern was recorded for subsequent analysis.

### 4.7. Promoter Self-Activation Detection and Yeast One-Hybrid Assay

For yeast one-hybrid assays, specific primers were designed (Table S7) to amplify the promoter fragments of *DobHLH68* and *DoPYL9* (pro*DobHLH68* and pro*DoPYL9*), which were subsequently cloned into the pHIS2 vector to construct recombinant plasmids pHIS2-pro*DobHLH68* and pHIS2-pro*DoPYL9*. These plasmids were transformed into *Saccharomyces cerevisiae* strain Y187 yeast competent cells (Weidi Biotechnology Co., Shanghai, China) following the manufacturer’s protocol. Well-growing colonies were selected, dissolved in ddH_2_O, and serially diluted to 10–1, 10–2, 10–3, 10–4, and 10–5. Then, 10 µL of the diluted cell suspension was spread onto SD/-T/-H medium containing 3-AT at concentrations of 0, 20, 40, 60, 80, 100, 120, 140, 160, and 180 mM (Coolaber Biotechnology Co., Ltd., Beijing, China) to assess the resistance of pHIS2-proDobHLH68 and pHIS2-proDoPYL9 to 3-AT. The most suitable concentration for 3-AT during the yeast one-hybrid assay was determined based on optimal growth (Figure S1).

The coding sequences of *DoDof15*, *DoDof22*, and *DoDof24* were amplified and inserted into the pGADT7 vector to generate effector constructs pGADT7-DoDof15, pGADT7-DoDof22, and pGADT7-DoDof24. The plasmids pHIS2-pro*DobHLH68* and pHIS2-pro*DoPYL9* were co-transformed with three effector vectors into Y187 yeast competent cells using the lithium acetate method (Weidi Biotechnology Co., Shanghai, China). Transformant strains were initially selected on SD/-Trp/-Leu (SD/-T/-L) medium, and positive clones were transferred onto SD/-Trp/-Leu/-His (SD/-T/-L/-H) medium containing 3-AT. Plates were incubated at 29 °C for 3–4 days, and yeast growth was monitored and recorded. Negative controls consisted of yeast co-transformed with pHIS2-proDobHLH68 and pGADT7, or pHIS2-proDoPYL9 and pGADT7.

### 4.8. Statistical Analysis

The qRT-PCR experimental data were analyzed using SigmaPlot 15.0 software, with the results presented as the means ± standard deviation (SD) of three biological replicates. Statistical significance was determined by one-way analysis of variance (ANOVA) followed by Duncan’s multiple range test between pairs of groups. Two groups with significant differences (*p* < 0.05) are denoted by different letters, while two groups with non-significant differences are indicated by the same letter.

## 5. Conclusions

We identified Dof transcription factors in *D. officinale* for the first time. This is also the first exploration of the conserved domains, expression patterns, and potential biological functions of Dof transcription factors in orchids. The genome contains 28 *DoDof* genes in *D. officinale*, all of which possess the C2-C2 conserved domain characteristic of the Dof family. These genes exhibit diversity in both gene structure and expression patterns. The expression pattern analysis suggests that the *DoDof* genes are likely involved in plant development and response to stress conditions. We identified potential target genes of Dof transcription factors, discovering that the promoters of 13,811 genes contain three or more core sequences of 5′-(T/A)AAAG-3′. Yeast one-hybrid assays indicate that there may be diversity in the recognition and binding of these core sequences. We found that DoDof22 binds to the promoter of the ABA receptor protein *DoPYL9*, suggesting that DoDof22 is involved in the ABA signaling pathway, regulating the stress response in *D. officinale*. Furthermore, DoDof15 and DoDof24 could bind to the promoter of *DobHLH68* in yeast. Research indicates that the bHLH transcription factors primarily participate in the secondary metabolism of plants, which implies that DoDof may regulate the expression of *DobHLH68*, thereby influencing the synthesis of secondary metabolites and enhancing the plant’s stress resistance. 

## Data Availability

The data presented in this study are available in the insert article or Supplementary Materials here.

## Supplementary Materials

The following supporting information can be downloaded at: https://www.mdpi.com/article/10.3390/ijms26062671/s1.
