# Supplementary material for: Genome-Wide Identification and Functional Characterization of the Dof Family in Dendrobium officinale
Source: Int J Mol Sci. 2025 Mar 16;26(6):2671. doi: 10.3390/ijms26062671 (PMC11942446; doi:10.3390/ijms26062671)
Supplement: Supplementary file 1 [file ijms-26-02671-s001.zip › ijms-3514705-supplementary.pdf]

Table S1. Physical and chemical characteristics of DoDof genes in *D. officinale*.

Gene name	Gene ID	Chromosome location	CDS (bp)	Protein length (aa)	Molecular Weight (kDa)	Theoretical pI
DoDof1	Dca025691	388:192140~192469	330	109	12.08	8.33
DoDof2	Dca001177	716:7943839~7944915	1077	358	37.33	8.59
DoDof3	Dca001545	716:19825825~19828152	798	265	29.62	9.12
DoDof4	Dca002115	133:11595310~11597206	768	255	27.24	9.42
DoDof5	Dca002782	231:4298276~4299121	846	281	29.97	8.11
DoDof6	Dca004946	847:5048144~5050341	921	306	34.95	9.37
DoDof7	Dca005129	1608:2434946~2436433	771	256	27.68	5.87
DoDof8	Dca005137	1608:2519631~2520570	633	210	22.46	5.37
DoDof9	Dca005366	370:2441681~2444793	672	223	24.03	8.49
DoDof10	Dca005933	743:1028958~1030950	1011	336	35.58	8.9
DoDof11	Dca006714	1022:1110551~1111333	783	260	27.97	8.38
DoDof12	Dca009280	488:1262459~1263441	552	183	19.86	7.64
DoDof13	Dca009868	1638:751031~752988	843	280	30.58	8.98
DoDof14	Dca010173	993:604365~605240	876	291	32.32	5.17
DoDof15	Dca011651	659:1813890~1814327	438	145	15.95	9.19
DoDof16	Dca012001	898:1337052~1339877	1272	423	46.64	6.8
DoDof17	Dca013032	412:172000~173471	657	218	23.48	9.67
DoDof18	Dca016494	292:333336~335885	933	310	32.44	9.08
DoDof19	Dca017041	1074:1029~1496	468	155	17.64	9.15
DoDof20	Dca018076	51:537299~538114	816	271	29.42	8.93
DoDof21	Dca018155	1448:209692~225907	1551	516	55.85	6.38
DoDof22	Dca018494	1034:362821~363755	663	220	25.03	8.87
DoDof23	Dca018925	1414:577691~582253	1455	484	52.54	6.39
DoDof24	Dca020537	1013:136340~137011	672	223	24.43	9.07
DoDof25	Dca020539	1013:152664~153495	600	199	21.65	9.59
DoDof26	Dca020928	1573:592194~593021	828	275	29.26	8.9
DoDof27	Dca021458	484:140389~141543	807	268	28	9.34
DoDof28	Dca023201	1508:395634~400586	789	262	29.24	8.93

Table S2. Gene information and amino acid sequences used to build phylogenetic trees.

Gene name	Gene ID	Amino acid sequences
DoDof1	Dca025691	MESVRDSSGFKLFGAVIATDKRTLAAAPEDDDVATEAAGEEEVDVAASLPCPRCKSQETKFCYFNNYNVNQPR<br>HFCKACHRYWTAGGTLRNVPVGAGRRRNRIAPPELR
DoDof2	Dca001177	MIQELLSAVVEERKFSFPGGGRLVSDDAPSPFLYSTSTATTLSPSSSSSTIQPSSSPSPSTASEPPQQKLRCPRCDSSNT<br>KFCYYNNYNLTQPRHFCKTCRRYWTKGGALRNVPIGGGCRKAKPVPIVAGSGKSGGGSKPNKQIPSLDAIARA<br>GFGFEPELFQPSPILWTGPTAPTPPPHTSHLLALLRAGGGAAIRSFEPSPLSSLSSIRIKEEGMMAEPTGVGLGLNG<br>LNLDPLGQFSSAAGMGSLWRNEGQMPYLQLQENGSPFGDAAAAAGIQELYQKLRPQAYCNDQLSIAMSNGW<br>SAPGCSSLATSATLTSSIGGGGGSSNSAVAGAEPAMGGEFGYWNPAFSSWSDMSSTN
DoDof3	Dca001545	METVQWPQQGVERKVRPHREQALNCPRCKSTNTKFCYYNNYSLSQPRYFCKSCRRYWTEGGSLRNVPVGGGS<br>RKNNKRLNSSSSSSSQASSKITTLATPTSSTSLLSSTSMNQQPSKLIPLELTSFSLSSSVSALQPIRSGIASRKFISFELPN<br>SSKENELSANARIRLRKSNPFQEDLHALSMPTIEDFGLQELMWPSKLKVPLDYGVGSMEEGKERRDGNLPFPFE<br>DLKQVSIKNACDENTVEGVDQTWFWEGIMGEGEGRGG
DoDof4	Dca002115	MAERARLAKLPQVEAALKCPRCDSTNTKFCYFNNYSLTQPRHFCKTCRRYWTRGGALRSVPVGGGCRRNKRG<br>NKSSSCKPSAPAPITPSASTSSAAAGVFRTAPPQFLAPWHHLPDYGFPHGIQQPIDHTMQENMGLEQWRIQQFP<br>FLGGLVEPSLPTAAMVPGLYPFEGELGGGHGKIMSTVKMEESSQAAAAAAATAATTAANMPRQFFNVGRNDG<br>QIWPGGGDSGSGEGHGGRWISGEHLSGFSSSSTGNLL
DoDof5	Dca002782	MANHQLDHEVLNCPKSSSSTSPAHHLQQEIKKPRPQPEQALRCPRCDSTNTKFCYYNNYSLSQPRYFCKGCRRY<br>WTKGGSLRNVPVGGGCRKNKRSSPSTSNSSSTSKKPALQDNQDQVNPTSLLIPSMPLPPPLTYDPSDLTLAFSSFH<br>SHDPFLLGNPNPNPNPNPNGAAFLDILRGGDHSQNHGGGGGGLHSFYYGFSGGGDDIRGMLSFDGGVGGCSD<br>STTVAAETGAVVGNSDHPASNAHSTSTSHGSLGMESGRDYWNGVPSNSSWHGLINSSLL
DoDof6	Dca004946	MDPVIKLFGKTIQLPAARQGLAATAGEDRSGYEKKFSPTILRASSKKQGNHEPKHKNEETRFSQQEERIDEKNMS<br>EAKAEKNNPMDKTLKKPDKILPCPRCKSMDTKFCYFNNYNVNQPRHFCKNCQRYWTEGGSMRNVPVGAGRR<br>KSKSSTTNQYYNHHIRIPDCAQADFYESNNHPSPLKHNGTVLSFSAEAPLLESMDSNLENQFSRSLATPWPNPW<br>SQTPLYPMAPYLAMPWLCSCRHSTVPKRLRITEDHEKAAKCSVWEKLGFKNDKTDKIFQTRSAEASQVLCANP<br>AALSRSLNFQERS
DoDof7	Dca005129	MGLSSNQVSVDIHHWPQGGLEFLKSEGREEKQNAPLLMCPRCESTNTKFCYYNNYSRSQPRHFCRVCRRHWTE<br>GGTLRNIPVGGGRKNKRRKITPTATSLAAAAGGNPIAPVVGEMKDPIFPDILRQVLLHQPPPPPPPLPLESPCMD<br>TLEGLFGASLPKTFLTFMEDFSGVSSGLALSAGEIPFMGAASTSHQNLSGVQDVSGGCGDGFLGMMDASAASIW<br>VKKETVFPASYWDFWDGGAEDLNIGAVAETMLPE

DoDof8	Dca005137	MCPRCESTNTKFCYYKGISNSQPRHFCRACRRHWTEGGTLRNIPVGGGCKNKRRKITPTATSFAAAAGGNPIAP<br>VVGELKDPIFPDILRHVLLNQPPQPPLESPSMDTMEGLFDAALSKSSLTFMEDFSGVSSGLAAASTSHQNLSGVQ<br>DVSGGCGDGFLGMMDASAASIWVKKETVFPASYWDFWDGGAEDLNIGAVAEEIVAETLLPE
DoDof9	Dca005366	MQGTTMTAFMASRPPLTEPEQNLPCPRCESTNTKFCYYNNYNLSQPRHFCKDCRRYWTKGGTLRNVPVGGGT<br>RKNSKRSLCSSGAASPSASGGVSSKRPSPSSSAGEVKNSELFSSSVPTVDNDHRMLDMTGSFSSLLSSTAQFGNFFE<br>CFQSLDSLPILKGVTRPMREPEAQSPAESSGNESAAAAPPTVMPENFLSLPGDTSSWAGGWPDLSIYTPGRNFQ
DoDof10	Dca005933	MVFSSLPIYLDPPNWSQQTSHQPPSTTTISHPPQLSSLMADPPPHPIGLPGPTRPVSMAERARLAKIPQPEPALKCP<br>RCDSTNTKFCYFNNYSLSQPRHFCKTCRRYWTRGGALRNVPVGGGCRRNKRTKAGSTSSKTSFSAGASSSTATA<br>SMNSTTILPPPPPPQLPFMTHFQPLSDYSTTNIGLGFTGIHPLDSVDYQIGSSGGGGVGLEQWRMQQMQQFPFM<br>GGLDASSTAPATVAGLFPFDGESSGNDHQGYSTGRMLTKASSSGIISQLASVKMEDSSHGINLQRQYLGVTGND<br>QYWDGNGDGGASVGNGGGSGWLGDLSGFNSSSSGNIL
DoDof11	Dca006714	MVAKLDSGTAAAQQNAGSPPLNCPRCDSTNTKFCYYNNYSLAQPRHFCKSCKRYWTRGGTLRNVPVGGGCR<br>KNKRTKKSHRSAPSITAAAPSLPPPPVIHSPATDLSSLLYGFPQMMNSSTVFFPKFELDAQFAGYEENFQSCLSAA<br>NIVDLQKSAAAPADFLGDYSLLNSHIPSSSSPMAAEAYQPLLSYDDINMGTENNTSNRSINNWQDTKGNPLEAT<br>NSFAASGGSGAVAQTPYLYWNQAMCSGWPESSSSVAPLI
DoDof12	Dca009280	MQGTTSASATATKSPFSDPEQNLQCPRCESTNTKFCYFNNYNLSQPRHFCKDCRRYWTRGGALRNVPVGGGTR<br>KNSKRSSATSAVAATSAVNPKRPLPSSSTGEAQRSELLPAPFTPVDDDHRMLDITGSFSSLLSSTNHLGNFLDGEA<br>ATPALAESFLGLPVDSSSWGWPDLTIYTPDTRFE
DoDof13	Dca009868	MDAQWTTQGIGVVKEVEISNTRQPQLQAEKRVRPYKEQSLNCPRCNSTNTKFCYYNNYSLSQPRYFCKTCRRY<br>WTEGGSLRNVPVGGGSRKNKRSSLANNSSSNPTTMTTSLVSLTSPNLQPTEPIPTVQIPIASSKFYQGSGQDLNLSF<br>QNQSLAHHDQLGAASGSISAMELLRSGISAKGFAPFPAAPGSVFGSGFGFQELMRQSNLKLPLDHHGRFSGSM<br>HQMQEGGGGRLLFPFEDLKQVHVNNGGHEEEDNGVVQEQGDPGLFWNGANGRGGGTW
DoDof14	Dca010173	MFSPEDHRMLSYATRSAPVDHRRWKPSLEIAPNCPRCDSSNTKFCYYNNYSLTQPRYFCKGCRRYWTKGGSLR<br>NVPVGGGCRKNRRGKSARLSSDSITSSTSSSSSLRIDTTRVLDSGLRPDQALDDMFDTYYPSSDSGGSNIDMAVMY<br>ARYLNQVPDKLPVEIDDSFGFIGSVTAELSSSSTPSTDMNCQTVSQADEGNLGFMASNCGMEESVVFPRLEYSSS<br>MLPEMNGGDVFSMNWGSVYPNMPWPAQEQNLAVASTRVQQEIGSNHHFQELVVGDWSSMDQSGFEAF
DoDof15	Dca011651	MADIGEEAPAFKLFGTVIVADENRLKKNSQPPPPLSSSEAAAAELPCPRCRSQETKFCYFNNYNVNQPRHFCKA<br>CHRYWTAGGALRNVPVGAGRRRSRFYARTGGETAAEGGRVDFTAEVERWLLRREQMPAAGGKLSNAASESC
DoDof16	Dca012001	MAESRDPAIKLFGKTIQIPVGVVVSLTIGEEFIGDDEKAPSDKEASPEQMDNNEEAPISREEEKKNDESNNLANEE<br>KIDQNSSSPENSNSKADDEQNESNNNPRDKANKKPDKILPCPRCKSMDTKFCYYNNYNVNQPRHFCKNCQRY<br>WTAGGSMRNVPVGAGRRKSKSSASHQHFRQITIADCVQAVPESIHHLQPLKSNGTVLSFGSDAPLCESMASVM<br>KLVEKTVQNRNQKEKEQLSGSPNLNEQVKASSTSNCYGFPPNVSNINGSPWPYTWTHPPFAFPFYPITAYWGM

		PWLSPPLSTSPSSGSSCSVSNSPNLGKHSREGNLLNHSHLDKGNTSIPEKSLWAPKTMRVDVPEKLQSVQCGQPLDSTLIKADLISRGGGLFKAFQSNGDHKNPSNELSQVLLANPAALSRSFTFQESS
DoDof17	Dca013032	MDAAQWSKEAAVAETAATKAPAISERKNRRKNDQKQLNCPRCNSNNTKFCYYNNYSLTQPRYFCKTCRRYWTEGGSLRNIPVGGGSRKNKRSITATSSSTPSSSSMQNHPKFSSTDQVRDLNLGFQQGGGSGLPELGILRGGGMSGRQMGSNFMHEVFGLQELGLGFQVGAGGATDHDEGRVLFSSSAPRDGILGQNRGLGGETQGFWNGMMGGGGSW
DoDof18	Dca016494	MDTVQWPQQGIGVVKGSVVETNQRQAQIQVERSRVRPHKEQSLNCPRCNSTNTKFCYYNNYSLTQPRYFCKTCRRYWTEGGSLRNVPVGGGSRKNKRSSSSSSSCSSSTSSNSTPTPTLTSTTPTTTLPSSSSTNLHPSEHMINHSLGSSKFYQGGSVHDLNLSFPNQSLGGHELGGSMSALELLRNGISARGLGPFVPAAMPPGPTSLFGAGFGFQELMRPASNSMKLHPLDGVGGSSGYGGSMQEEGDGRLLFPFEDLKQVSMSNGSHEGVVQGHGDPTLFWNGIMGSGGGGGGGNGGGGGAGGGASW
DoDof19	Dca017041	MFPIYSFHPLPTTDRRWRPKVEEVAPNCPRCDSPNTKFCYYNNYCLAQPRYLCKGCRRYWTKGGSLRSIPVGGGCRKTRRGKSAKVISTLSSSLPSPSNSFRPDQALENMSGRTGGNFLMSDQYLDQTPLSFTQEAFAQAHDFDTNLMEASREDY
DoDof20	Dca018076	MQDFQSITGQSTGRIFGGVTDGTGGGWDNRRLRAYPSATMAPSPLHPPPPLKCPRCESQNTKFCYYNNYNLSQPRHFCKSCRRYWTKGGVLRNVPVGGGCRKPKRSSNSKSRRSSASRSSSDSSGRNPADASAALPDSVIFPNSSSSNPNPPNPSFDQPFAAAPDRAAEIFPDGAGTSFSNLMAVTSSQSIHGFNFVNPPLLRHPDPHQKLEDITSQDIIHQVVPGRANNSAIDWPPAMDTSLYDLAGATDPTAYWSQNHWVDGDPSLYLP
DoDof21	Dca018155	MARVDSGGMSEVRDPAIKLFGRKIPLLEEQPLAEEGGVADLAQAMLPLKVGEKDTCKEKANTKVDAMLDAPENLDQNESLSSSGLCHGIEENQKTATEIAKDAEEPKSEQDNKESGTSGEDKVLKKPDKVLQCPRCNSMDTKFCYYNNYNVNQPRHFCKNCQRYWTAGGTMRNVPVGAGRRKNKHSSSTYRHTVVMPDALASVQVDGSDLSHHEASPCGSSTTTRLPLKCNGTVLKFGPEAPLCESMTSVLKLGDQKRASELGFTTHRENKEEPSCSSSAKVSKHAENGFPEDAEQKALHGYQNGLAPLHHLQCYPVSPWAYAWNPGWSNIAAAAAMAAAGRSPEPLPRQENGIPTPISWSRPPMVTAPPFCAPTFPFPLVPTSFWSCIPSWTGGPWNLPLVGNNNRLSPSSSTSNSNCSGNGSPTLGKHCRDATSIGEENTEKSLWVPKTLRIDDIDEAAKSSIFATLGIKLDEGCLFKAFPSKSQKGEAADAAQLLHANPAAFSRSQSFQETTT
DoDof22	Dca018494	MLSIYSFHPLPTTDRRWRPKIEEVGPNCPGCDSQNTKFYYYNNYCLAQPRYLCKGCRRYWTKGGSLRSIPVGGGFRENHRGKSAKFISTLSSSLPSPPNSFRPDKALENMSGSTSGTFLLPDQYLDQLPELPQQEALPKPMMKQFNGGSRIEIRAEAVEKINFWHDEVMEKEGRGAEGVMHHNVCLMGSSNYGFQTNLMWESARFQWIGISKMMSLEASL
DoDof23	Dca018925	MSEGRDPAIKLFGRKIPLQEGQTRMDEGDVADSGSAAEPPPPPLEEKDTCKEKAKIEVANKLGAPGELDPKKALDSSVLNNSSVVVEESSTESAKPVEETQPEQDHKEADIAGQDKLLKKPDKLLPCPRCNSMDTKFCYYNNYNVNQPRHFCKNCQRYWTAGGSMRNVPVGAGRRKNKHTGSGYRHTVITPDSLPSVQVDGPDLIDPKPLSPFKVNGTILKFGPEAPLCDSMASILNLGEQNLASQLDFTTGAENREETSCSSACKPKENGFPEKTQQNPMNSYCNGITPLPQIQ

		YFPGPPWASHWNPGWNNFAAAMAATGCSPELLPVQDNGILWHQPALITSSFCPHPPLPFPFISPSFWGWTGGP WNLPWLGSSNGVSLSSSKSTSYCSGNGSPTLGKHSREEKTEKSLWIPKTLRIDDPDEAAKSSIFATLGIKHDEVSKF KAFSSSNGNGKGDSSKTALALHANPAALSRSRSFQEIIS
DoDof24	Dca020537	MAANIYPNVHSTATAAIAGGTPALQNSATPHLKCPRCDSTNTKFCYYNNYSLAQPRHFCRTCKRYWTRGGTL RDVPIGGGCRKHKRHNKPKSAIVAALPNSPTLRQLANPLRPPAADLSSLILRMPPATLSNRFFPMFEEAIQSGIES HHINTLQQSAEAVKSYQAPLLCYDDLPTAICEGLSLIKENPTEGIYSFNGYSSAAAESAYLYAWPESCSSVVRLL
DoDof25	Dca020539	MEQRHHQLDKQGSRNMASESKSTSEPPRNCPRCESTNTKFCYYNNYSSSQPRYFCRACRRYWTQGGALRNLPH GSSATRRVAKQSKRPSPTSYYSVAVSTTSVTPLYHQMNTGTVEPPRCFSGGRLPRQLSMGSQTIDPSMVVGVRIPP AQPEMAGAVSTVAGEEGDRILGNLFGGFEDGSGGEGAVKKMPSASSGPFI
DoDof26	Dca020928	MASDSKAAATKSSGCGGGGTGASAGLRLPEQGLKCPRCDSPNTKFCYYNNYSLTQPRHFCKSCRRYWTKGGA LRNVPVGGGCRKNRKSKSSSSSASSSSSSLLPLNPEPGKALTQTAMDLFLPFSYGANTAASMAGFSYNGRNSNYG SIASSIESLSSINQDLHWRLQQQRMQFLFGGELAQKDNIVSFMNSQVVEPNCFGMAENNKGEIFGDSSGANGSR KGSETGTAWFLDGSFAMPAPSPINTDVDMNNPNSVSDWNGISGWSDVHNFTALP
DoDof27	Dca021458	MVFPSLPLYLDPPNSWNQPPPTHQPVGAGATTSDEHQPPPPAATTGAGTSRPGSMAERARLAKIPQPEAALSCP RCDSNNTKFCYFNNYSLSQPRHFCKTCRRYWTRGGALRNVPVGGGCRRNKRGGKSSSLTSTSTQPKLQPSKSPT STTATSAPPANLVLLSAAPPLSLLAAAPWNQLQEYGYAGQGLEQWRLPYLGGMEVPAAAGMQVPAPAAAAV TAGMYGYHEGEWSVGGGINGNVGGWMSNSITDHHALGFSGSSSAGNGNL
DoDof28	Dca023201	MRQRKEREKAKDCAKMEISNANEQQVMSSHGLEELFIACRKPPAPALEKKTRTQPEQALRCPRCDSTNTKFCY YNNYSLSQPRYFCKGCRRYWTKGGSLRNVPVGGGCRKNKRSSSSSSLSSSSSSSSNSIIPSSEKPQDDQTSFISYSHG ELTLAFSRQVWPSFADHERFFLGNPNPNSGSSGGYLNFMRNGFLETSNINGYHQINYGNNGSVVEEVEGNNPN TTERSCKMLMDIQMSNGVDGSLGMEYSSWHGIINSSLI
AT1G07640	AT1G07640	MGGSMAERARQANIPPLAGPLKCPRCDSSNTKFCYYNNYNLTQPRHFCKGCRRYWTQGGALRNVPVGGGCR RNNKKGKNGNLKSSSSSSKQSSSVNAQSPSSGQLRTNHQFPFSPTLYNLTQLGGIGLNLAATNGNNQAHQIGSS LMMSDLGFLHGRNTSTPMTGNIHENNNNNNNNNENNLMASVGSLSPFALFDPTTGLYAFQNDGNIGNNVGISGS STSMVDSRVYQTPPVKMEEQPNLANLSRPVSGLTSPGNQTNQYFWPGSDFSGPSNDLL
AT1G21340	AT1G21340	MLPYIGHNSYQQHQFPLPEMEIPEKWKLSYEQEAITAPACPRCASSNTKFCYYNNYSLSQPRYFCKGCRRYWTK GGSLRNIPVGGGCRKRSRSRQNSHKRFGRNENRPDGLINQDDGFQSSPPGSDIDLAAVFAQYVTDRSPSSTDNTT GSDQDSPITTTTHALESLSWDICQETDVDLGFYGEFNNLTQKTKEDQEVFGQFLQEDREEIFEFQGLLDDKEIQEI LECSFSEEPDQLVSQGSFMINGDNWSSTDLTRFGI
AT1G26790	AT1G26790	MWLSHLFMSLSKLTCNFSIFSVFMACGSIGMSQVRDTPVKLFGWTITPVSHDPYSSSSHVLPDSSSSSSSSLSLRPH MMNNQSVTDNTSLKLSSNLNNESKETSENSDDQHSEITTITSEEEKTTELKKPDKILPCPRCNSADTKFCYYNNY NVNQPRHFCRKCQRYWTAGGSMRIVPVGSGRRKNKGWVSSDQYLHITSEDTDNYNSSSTKILSFESSDSLVTERP KHQSNEVKINAEPVSQEPNNFQGLLPPQASPVSPPWPYQYPPNPSFYHMPVYWGCAIPVWSTLDTSTCLGKRTR

		DETSHETVKESKNAFERTSLLLESQSIKNETSMATNNHVWYPVPMTREKTQEFSFFSNGAETKSSNNRFVPETYL<br>NLQANPAAMARSMNFRESI
AT1G28310	AT1G28310	MQSKNMIVASSHQQQQQQPQQPQPQLKCPRCDSSNTKFCYYNNYSLSQPRHFCKACKRYWTRGGTLRNVP<br>VGGSYRKNKRVKRPSTATTTASTVSTTNSSSPNNPHQISHFSSMNHHPLFYGLSDHMSSCNNNLPMIPSRFSDS<br>SKTCSSSGLESEFLSSGFSSLSALGLGLPHQMSHDHTINGSFINNSTTNKPFLLSGLFGSSMSSSSTLLQHPHKPMN<br>NGGDMLGQSHLQTLASLQDLHVGGNNEDMKYKEGKLDQISGNINGFMSSSSSLDPSNYNNMWNNASVVNG<br>AWLDPTNNNVGSSLTSLI
AT1G29160	AT1G29160	MATQDSQGIKLFGKTITFNANITQTIKKEEQQQQQPELQATTAVRSPSSDLTAEKRPDKIIPCPRCKSMETKFCY<br>FNNYNVNQPRHFCKGCQRYWTAGGALRNVPVGAGRRKSKPPGRVGGFAELLGAATGAVDQVELDALLVEE<br>WRAATASHGGFRHDFPVKRLRCYTDGQSC
AT1G47655	AT1G47655	MPSEPNQTRPTRVQPSTAAYPPPNLAEPLPCPRCNSTTTKFCYYNNYNLAQPRYYCKSCRRYWTQGGTLRDVP<br>VGGGTRRSSSKRHRSFSTTATSSSSSSSVITTTTQEPATTEASQTKVTNLISGHGSFASLLGLGSGNGGLDYGFGYG<br>YGLEEMSIGYLGDSSVGEIPVVDGCGGDTWQIGEIEGKSGGDSLIWPGLEISMQTNDVK
AT1G51700	AT1G51700	MQDLTSAAAYYHQSMMMTTAKQNQPELPEQEQLKCPRCDSPNTKFCYYNNYNLSQPRHFCKNCRRYWTKG<br>GALRNIPVGGGTRKSNKRSGSSPSSNLKNQTVAEKPDHHGSGSEEKEERVSGQEMNPTRMLYGLPVGDPNGAS<br>FSSLLASNMQMGGLVYESGSRWLPGMDLGLGSVRRSDDTWTDLAMNRMEKN
AT1G64620	AT1G64620	MDTAKWPQEFVVKPMNEIVTNTCLKQQSNPPSPATPVERKARPEKDQALNCPRCNSLNTKFCYYNNYSLTQPR<br>YFCKDCRRYWTAGGSLRNIPVGGGVRKNKRSSSNSSSSSPSSSSSSKKPLFANNNTPTPPLPHLNPKIGEAAATKV<br>QDLTFSQGFGNAHEVKDLNLAFSQGFGIGHNHHSSIPEFLQVVPSSSMKNNPLVSTSSSLELLGISSSSASSNSRPA<br>FMSYPNVHDSSVYTASGFGLSYPQFQEFMRPALGFSLDGGDPLRQEEGSSGTNNGRPLLPFESLLKLPVSSSSTNS<br>GGNGNLKENNDEHSDHEHEKEEGEADQSVGFWSGMLSAGASAAASGGSWQ
AT1G69570	AT1G69570	MSKSRDTEIKLFGRTITSLLDVNCYDPSSLSPVHDVSSDPSKEDSSSSSSSCSPTIGPIRVPVKKSEQESNKFKDPYILS<br>DLNEPPKAVSEISSPRSSKNNCDQQSEITTTTTTSTTSGEKSTALKKPDKLIPCPRCESANTKFCYYNNYNVNQPR<br>YFCRNCQRYWTAGGSMRNVPVGSGRRKNKGWPSSNHYLQVTSEDCDNNNSGTILSFGSSESSVTETGKHQSGD<br>TAKISADSVSQENKSYQGFLPPQVMLPNNSSPWPYQWSPTGPNASFYPVPFYWGCTVPIYPTSETSSCLGKRSRD<br>QTEGRINDTNTTITTTRARLVSESLRMNIEASKSAVWSKLPTKPEKKTQGFSLFNGFDTKGNSNRSSLVSETSHSL<br>QANPAAMSRAMNFRESMQQ
AT2G28510	AT2G28510	MDPEQEISNETLETILVSSTKGSNNNNKKMEEEMKKKVSRGELGGEAQNCPRCESPNTKFCYYNNYSLSQPRYF<br>CKSCRRYWTKGGTLRNVPVGGGCRRNKRSSSSAFSKNNNNKSINFHTDPLQNPLITGMPPSSFGYDHSIDLNLA<br>FATLQKHHLSSQATTPSFGFGGDLSIYGNSTNDVGIFGGQNGTYNNSLCYGFMSGNGNNNQNEIKMASTLGMS<br>LEGNERKQENVNNNNNNSENPSKVFWGFPWQMTGDSAGVVPEIDPGRESWNGMVSSWNNGLLNTPLV
AT2G28810	AT2G28810	MVFSSVSSFLDPPINWPQSANPNNHPHHHQLQENGSLVSGHHQVLSHHFPQNPNPNHHHVETAAATTVDPSS<br>LNGQAAERARLAKNSQPPEGALKCPRCDSANTKFCYFNNYNLTQPRHFCKACRRYWTRGGALRNVPVGGGC

		RRNKKGKSGNSKSSSSSQNKQSTSMVNATSPTNTSNVQLQTNSQFPFLPTLQNLTQLGGIGLNLAAINGNNGG NGNTSSSFLNDLGFFHGGNTSGPVMGNNNENNLMTSLGSSSHFALFDRTMGLYNFPNEVNMGLSSIGATRVSQ TAQVKMEDNHLGNISRPVSGLTSPGNQSNQYWTGQGLPGSSSNDHHHQHLM
AT2G34140	AT2G34140	MATQDSQGIKLFGKTIAFNTRTIKNEEETHPPEQEATIAVRSSSSSDLTAEKRPDKIIACPRCKSMETKFCYFNNYN VNQPRHFCKGCHRYWTAGGALRNVPVGAGRRKSKPPGRVVVGMLGDGNGVRQVELINGLLVEEWQHAAA AAHGSFRHDFPMKRLRCYSDGQSC
AT2G37590	AT2G37590	MVFSSIQAYLDSSNWQQAPPSNYNHDGTGASANGGHVLRPQLQPQQQPQQQPHPNGSGGGGGGGGGSIRAG SMVDRARQANVALPEAALKCPRCESTNTKFCYFNNYSLTQPRHFCKTCRRYWTRGGALRNVPVGGGCRRNRR TKSNSNNNNNNSTATSNNTSFSSGNASTISTILSSHYGGNQESILSQILSPARLMNPTYNHLGDLTSNTKTDNNMS LLNYGGLSQDLRSIHMGASGGSLMSCVDEWRSASYHQQSSMGGGNLEDSSNPNPSANGFYSFESPRITSASISSA LASQFSSVKVEDNPYKWVNVNGNCSSWNDLSAFGSSR
AT2G46590	AT2G46590	MMNVKPMEQIMIPNNNTHQPNTTSNARPNTILTSNGVSTAGATVSGVSNNNNNNTAVVAERKARPQEKLNCP RCNSTNTKFCYYNNYSLTQPRYFCKGCRRYWTEGGSLRNVPVGGSSRKNKRSSSSSSSNILQTIPSSLPDLNPPILF SNQIHNKSKGSSQDLNLLSFPVMQDQHHHHVHMSQFLQMPKMEGNGNITHQQQPSSSSSVYGSSSSPVSALEL LRTGVNVSSRSGINSSFMPSGSMMDSNTVLYTSSGFPTMVDYKPSNLSFSTDHQGLGHNSNNRSEALHSDHHQ QGRVLFPFGDQMKELSSSITQEVDHDDNQQQKSHGNNNNNNNNSSPNNGYWSGMFSTTGGGSSW
AT3G21270	AT3G21270	MQDPAAYYQTMMAKQQQQQQPQFAEQEQLKCPRCDSPNTKFCYYNNYNLSQPRHFCKSCRRYWTKGGALR NVPVGGGSRKNATKRSTSSSSSASSPSNSSQNKKTKNPDPDPDPRNSQKPDLDPTRMLYGFPIGDQDVKGMEIG GSFSSLLANNMQLGLGGGGIMLDGSGWDHPGMGLGLRRTEPGNNNNNPWTDLAMNRAEKN
AT3G45610	AT3G45610	MDYSSMHQNVMGVSSCSTQDYQNQKKPLSATRPAPPEQSLRCPRCDSTNTKFCYYNNYSLSQPRYFCKSCRRY WTKGGILRNIPIGGAYRKHKRSSSATKSLRTTPEPTMTHDGKSFPTASFGYNNNNISNEQMELGLAYALLNKQP LGVSSHLGFGSSQSPMAMDGVYGTTSHQMENTGYAFGNGGGGMEQMATSDPNRVLWGFPWQMNMGGGSG HGHGHVDQIDSGREIWSSTVNYINTGALL
AT3G47500	AT3G47500	MMMETRDPAIKLFGMKIPFPSVFESAVTVEDDEEDDWSGGDDKSPEKVTPELSDKNNNNCNDNSFNNSKPETL DKEEATSTDQIESSDTPEDNQQTTPDGKTLKKPTKILPCPRCKSMETKFCYYNNYNINQPRHFCKACQRYWTAG GTMRNVPVGAGRRKNKSSSSHYRHITISEALEAARLDPGLQANTRVLSFGLEAQQQHVAAPMTPVMKLQEDQ KVSNGARNRFHGLADQRLVARVENGDDCSSGSSVTTSNNHSVDESRAQSGSVVEAQMNNNNNNNNMNGYAC IPGVPWPYTWNPAMPPPGFYPPPGYPMPFYPYWTIPMLPPHQSSSPISQKCSNTNSPTLGKHPRDEGSSKKDNET ERKQKAGCVLVPKTLRIDDPNEAAKSSIWTTLGIKNEAMCKAGGMFKGFDHKTKMYNNDKAENSPVLSANPA ALSRSHNFHEQI
AT3G50410	AT3G50410	MPTSDSGEPRRIAMKPNGVTVPISDQQEQLPCPRCDSSNTKFCYYNNYNFSQPRHFCKACRRYWTHGGTLRDV PVGGGTRKSAKRSRTCSNSSSSSVSGVVSNSNGVPLQTTPVLFPQSSISNGVTHTVTESDGKGSALSLCGSFTSTLL

		NHNAAATATHGSGSVIGIGGFGIGLGSGFDDVSFGLGRAMWPFSTVGTATTTNVGSNGGHHAVPMPATWQFE GLESNAGGGFVSGEYFAWPDLSITTPGNSLK
AT3G52440	AT3G52440	MERAEALTSSFIWRPNANANAEITPSCPRCGSSNTKFCYYNNYSLTQPRYFCKGCRRYWTKGGSLRNVPVGGGC RKSRRPKSSSGNNTKTSLTANSGNPGGGSPSIDLALVYANFLNPKPDESILQENCDLATTDFLVDNPTGTSMDPS WSMDINDGHHDHYINPVEHIVEECGYNGLPPFPGEELLSLDTNGVWSDALLIGHNHVDVGVTPVQAVHEPVV HFADESNDSTNLLFGSWSPFDFTADG
AT3G55370	AT3G55370	MVFSSLPVNQFDSQNWQQQGNQHQLECVTTDQNPNNYLRQLSSPPTSQVAGSSQARVNSMVERARIAKVPLP EAALNCPRCDSTNTKFCYFNNYSLTQPRHFCKTCRRYWTRGGSLRNVPVGGGFRRNKRSKSRSKSTVVVSTDNT TSTSSLTSRPSYSNPSKFHSYGQIPEFNSNLPILPPLQSLGDYNSSNTGLDFGGTQISNMISGMSSSGGILDAWRIPPS QQAQQFPFLINTTGLVQSSNALYPLLEGGVSATQTRNVKAEENDQDRGRDGDGVNNLSRNFLGNININSGRNE EYTSWGGNSSWTGFTSNNSTGHLSF
AT3G61850	AT3G61850	MDATKWTQGFQEMINVKPMEQMISSTNNNTPQQQPTFIATNTRPNATASNGGSGGNTNNTATMETRKARPQ EKVNCPRCNSTNTKFCYYNNYSLTQPRYFCKGCRRYWTEGGSLRNVPVGGSSRKNKRSSTPLASPSNPKLPDLN PPILFSSQIPNKSNKDLNLLSFPVMQDHHHHALELLRSNGVSSRGMNTFLPGQMMDSNSVLYSSLGFPTMPDYK QSNNNLSFSIDHHQGIGHNTINSNQRAQDNNDDMNGASRVLFPFSDMKELSSTTQEKSHGNNTYWNGMFSN TGGSSW
AT4G00940	AT4G00940	MDHHQYHHHDQYQHQMMTSTNNNSYNTIVTTQPPPTTTTMDSTTATTMIMDDEKKLMTTMSTRPQEPRNCP RCNSSNTKFCYYNNYSLAQPRYLCKSCRRYWTEGGSLRNVPVGGGSRKNKKLPFPNSSTSSSTKNLPDLNPPFVF TSSASSSNPSKTHQNNNDLSLSFSSPMQDKRAQGHYGHFSEQVVTGGQNCLFQAPMGMIQFRQEYDHEHPKK NLGFSLDRNEEEIGNHDNFVVNEEGSKMMYPYGDHEDRQQHHHVRHDDGNKKREGGSSNELWSGIILGGDS GGPTW
AT4G21030	AT4G21030	MNNLNVFTNEDNEMNVMPPPRVCPRCYSDQTRFSYFNNNKKSQPRYKCKNCCRCWTHGGVLRNIPVTGICD KSNLPKIDQSSVSQMILAEIQQGNHQPFKKFQENISVSVSSSSDVSIVGNHFDDLSELHGITNSTPIRSFTMDRLDF GEESFQQDLYDVGSNDLIGNPLINQSIGGYVDNHKDEHKLQFEYES
AT4G21040	AT4G21040	MDNFNVVANEDNQVNDVKPPPPPPRVCARCDSDNTKFCYYNNYSEFQPRYFCKNCRRYWTHGGALRNVPIG GSSRAKRTRINQPSVAQMVSVGIQPGSHKPFFNVQENNDFVGSFGASSSSFVAAVGNRFSSLSHIHGGMVTNVH PTQTFRPNHRLAFHNGSFEQDYYDVGSDNLLVNQQVGGYVDNHNGYHMNQVDQYNWNQSFNNAMNMNY NNASTSGRMHPSHLEKGGP
AT4G21050	AT4G21050	MDNLNVFANEDNQVNGLKRPPPSRVCPRCDSDNTKFCFYNNYSESQPRYFCKNCRRYWTHGGALRNIPVGGS CRKPKRLKVDQSSISEMVSVENQPINHQSFRQTQENNEFVRSFDASSSATVTAVPNHFGYLSELHGVTNLLPIQSF RTMDCLDFGDESFQQGYYDVGSNDLIDNPLINQSIGGYVDNLTSYCINQVEPKLQPRYEHES
AT4G21080	AT4G21080	MDNLNVFANEDNQVNDVKPPPPPPRVCARCDSDNTKFCYYNNYCEFQPRYFCKNCRRYWTHGGALRNIPIG GSSRAKRARVNQPSVARMVSVETQRGNNQPFSNVQENVHLVGSFGASSSSSVGAVGNLFGSLYDIHGGMVTNL

		HPTRTVRPNHRLAFHDGSFEQDYYDVGSDNLLVNQQVGGYGYHMNPVDQFKWNQSFNNTMNMNYNNDST SGSSRGSDMNVNHDNKKIRYRNSVIMHPCHLEKDGP
AT4G24060	AT4G24060	MDTAQWPQEIVVKPLEEIVTNTCPKPQPQPLQPQQPPSVGGERKARPEKDQAVNCPRCNSTNTKFCYYNNYSL TQPRYFCKGCRRYWTEGGSLRNIPVGGGSRKNKRSHSSSDISNNHSDSTQPATKKHLSDHHHHLMSMSQQGL TGQNPKFLETTQQDLNLGFSPHGMIRTNFTDLIHNIGNNTNKSNNNNNPLIVSSCSAMATSSLDLIRNNSNNG NSSNSSFMGFPVHNQDPASGGFSMQDHYKPCNTNTTLLGFSLDHHHNNGFHGGFQGGEEGGEGGDDVNGR HLFPFEDLKLPVSSSSATINVDINEHQKRGSGSDAAATSGGYWTGMLSGGSWC
AT4G38000	AT4G38000	MMTSSHQSNTTGFKPRRIKTTAKPPRQINNKEPSPATQPVLKCPRCDSVNTKFCYYNNYSLSQPRHYCKNCRRY WTRGGALRNVPIGGSTRNKNKPCSLQVISSPPLFSNGTSSASRELVRNHPSTAMMMMSSGGFSGYMFPLDPNFN LASSSIESLSSFNQDLHQKLQQQRLVTSMFLQDSLPVNEKTVMFQNVELIPPSTVTTDWVFDRFATGGGATSGN HEDNDDGEGNLGNWFHNANNNALL
AT5G02460	AT5G02460	MVFSSFPTYPDHSSNWQQQHQPITTTVGFTGNNINQQFLPHHPLPPQQQQTPPQLHHNNGNGGVAVPGGPG GLIRPGSMAERARLANIPLPETALKCPRCDSTNTKFCYFNNYSLTQPRHFCKACRRYWTRGGALRSVPVGGGCR RNKRTKNSSGGGGGSTSSGNSKSQDSATSNDQYHHRAMANNQMGPPSSSSLSSLLSSYNAGLIPGHDHNSNN NNILGLGSSLPPLKLMPPLDFTDNFTLQYGAVSAPSYHIGGGSSGGAAALLNGFDQWRFPATNQLPLGGLDPFD QQHQMEQQNPGYGLVTGSGQYRPKNIFHNLISSSSSASSAMVTATASQLASVKMEDSNNQLNLSRQLFGDEQQ LWNIHGAAAASTAAATSSWSEVSNNFSSSSTSNI
AT5G39660	AT5G39660	MADPAIKLFGKTIPLPELGVVDSSSSYTGFLTETQIPVRLSDSCTGDDDDEEMGDSGLGREEGDDVGDGGGESET DKKEEKDSECQEESLRNESNDVTTTTSGITEKTETTKAAKTNEESGGTACSQEGKLKKPDKILPCPRCNSMETKF CYYNNYNVNQPRHFCKKCQRYWTAGGTMRNVPVGAGRRKNKSPASHYNRHVSITSAEAMQKVARTDLQHP NGANLLTFGSDSVLCESMASGLNLVEKSLLKTQTVLQEPNEGLKITVPLNQTNEEAGTVSPLPKVPCFPGPPPTW PYAWNGVSWTILPFYPPPAYWSCPGVSPGAWNSFTWMPQPNSPSGSNPNSPTLGKHSRDENAAEPGTAFDETE SLGREKSKPERCLWVPKTLRIDDPEEAAKSSIWETLGIKKDENADTFGAFRSSTKEKSSLSEGRLPGRRPELQANP AALSRSANFHESS
AT5G60200	AT5G60200	MDHLLQHQDVFGNYNKAREAMGLSYSSNPTPLDNDQKKPSPATAVTRPQPPELALRCPRCDSTNTKFCYYNN YSLTQPRYFCKSCRRYWTKGGTLRNIPVGGGCRKNKRSTSSAARSLRTTPEPASHDGKVFSAAGFNGYSNNEHI DLSLAFALLNKQHPGSSSQLGFHSELGSSHQSDMEGMFGTSQQKENATYAFGNGSSGLGDPSRVLWGFPWQM NGESFGMMNIGGGGGHVDQIDSGREMWTNMNYINSGALM
AT5G60850	AT5G60850	MQDIHDFSMNGVGGGGGGGGRFFGGGIGGGGGGDRRMRAHQNNILNHHQSLKCPRCNSLNTKFCYYNNYN LSQPRHFCKNCRRYWTKGGVLRNVPVGGGCRKAKRSKTKQVPSSSSADKPTTTQDDHHVEEKSSTGSHSSSESS SLTASNSTTVAAVSVTAAAEVASSVIPGFDMPNMKIYGNGIEWSTLLGQGSSAGGVFSEIGGFPAVSAIETTPFGF GGKFVNQDDHLKLEGETVQQQQFGDRTAQVEFQGRSSDPNMGFEPLDWGSGGGDQTLFDLTSTVDHAYWSQ SQWTSSDQDQSGLYLP

AT5G62430	AT5G62430	MLETKDPAIKLFGMKIPFPTVLEVADEEEEKNQNKTLTDQSEKDKTLKKPTKILPCPRCNSMETKFCYYNNYNV NQPRHFCKACQRYWTSGGTMRSVPIGAGRRKNKNNSPTSHYHHVTISETNGPVLSFSLGDDQKVSSNRFGNQK LVARIENNDERSNNNTSNGLNCFPGVSWPYTWNPAFYPVYPYWSMPVLSSPVSSSPTSTLGKHSRDEDETVKQK QRNGSVLVPKTLRIDDPNEAAKSSIWTTLGIKNEVMFNGFGSKKEVKLSNKEETETSLVLCANPAALSRSINFHE QM
AT5G62940	AT5G62940	MGLTSLQVCMDSDWLQESESSGGSMLDSSTNSPSAADILAACSTRPQASAVAVAAAALMDGGRRLRPPHDHP QKCPRCESTHTKFCYYNNYSLSQPRYFCKTCRRYWTKGGTLRNIPVGGGCRKNKKPSSSNSSSSTSSGKKPSNIVT ANTSDLMALAHSHQNYQHSPLGFSHFGGMMGSYSTPEHGNVGFLESKYGGLLSQSPRPIDFLDSKFDLMGVNN DNLVMVNHGSNGDHHHHHNHHMGLNHGVGLNNNNNNGGFNGISTGGNGNGGGLMDISTCQRLMLSNY DHHHYNHQEDHQRVATIMDVKPNPKLLSLDWQQDQCYSNGGGSGGAGKSDGGGYGNGGYINGLGSSWNG LMNGYGTSTKTNSLV
AT5G65590	AT5G65590	MSSHTNLPSPKPVPKPDHRISGTSQTKKPPSSSVAQDQQNLKCPRCNSPNTKFCYYNNYSLSQPRHFCKSCRRY WTRGGALRNVPIGGGCRKTKKSIKPNSSMNTLPSSSSSQRFFSSIMEDSSKFFPPPTTMDFQLAGLSLNKMNDLQL LNNQEVLDLRPMMSSGRENTPVDVGSGLSLMGFGDFNNNHSPTGFTTAGASDGNLASSIETLSCLNQDLHWRL QQQRMAMLFGNSKEETVVVERPQPILYRNLEIVNSSSPSSPTKKGDNQTEWYFGNNSDNEGVISNNANTGGGG SEWNNGIQAWTDLNHYNALP
AT5G66940	AT5G66940	MPSEFSESRRVPKIPHGQGGSVAIPTDQQEQLSCPRCESTNTKFCYYNNYNFSQPRHFCKSCRRYWTHGGTLRDI PVGGVSRKSSKRSRTYSSAATTSVVGSRNFPLQATPVLFPQSSSNGGITTAKGSASSFYGGFSSLINYNAAVSRNGP GGGFNGPDAFGLGLGHGSYYEDVRYGQGITVWPFSSGATDAATTTSHIAQIPATWQFEGQESKVGFVSGDYVA

Table S3. Prediction of subcellular localization of *DoDof* genes in *D. officinale*.

Gene name	Subcellular localization
DoDof1	nucleus
DoDof2	nucleus
DoDof3	nucleus
DoDof4	nucleus
DoDof5	nucleus
DoDof6	nucleus
DoDof7	nucleus
DoDof8	nucleus
DoDof9	nucleus
DoDof10	nucleus
DoDof11	nucleus
DoDof12	nucleus
DoDof13	nucleus
DoDof14	nucleus
DoDof15	nucleus
DoDof16	nucleus
DoDof17	nucleus
DoDof18	nucleus
DoDof19	nucleus
DoDof20	nucleus
DoDof21	nucleus
DoDof22	nucleus
DoDof23	nucleus
DoDof24	nucleus
DoDof25	nucleus
DoDof26	nucleus
DoDof27	nucleus
DoDof28	nucleus

Table S4. The promoter 1000 bp contains the amount of 5′-(T/A)AAAG-3′ in *D. officinale*.

5′-(T/A)AAAG-3′	Total quantity
≥ 1	27164
≥ 2	23491
≥ 3	18734
≥ 4	13811
≥ 5	9363
≥ 6	6084
≥ 7	3712
≥ 8	2064
≥ 9	1162
≥ 10	627
≥ 11	327
≥ 12	180
≥ 13	99
≥ 14	58
≥ 15	38
≥ 16	18
≥ 17	14
≥ 18	6

Table S5. Primers of qRT-PCR assay used for differential expression genes.

Primer name	Primer sequences (5′→3′)
DoDof1-RT-F	GCGGTGATTGCAACCGATAA
DoDof1-RT-R	AACTTCGTCTCCTGGCTCTT
DoDof2-RT-F	CTTCTTCCTCTCCATCACCATC
DoDof2-RT-R	GGCTGCGTGAGGTTATAGTT
DoDof3-RT-F	ACCTTCTAAGCTCATTCCTCTTG
DoDof3-RT-R	AAGCGATCCCACTCCTTATTG
DoDof4-RT-F	CACTCCATCTGCTTCCACTT
DoDof4-RT-R	TCCATGAGGAAATCCGTAATCC
DoDof5-RT-F	GGTGGAGGTTGCAGAAAGAA
DoDof5-RT-R	GTAGAGGCATGGAAGGAATGAG
DoDof6-RT-F	ATGGAACAGTACTCAGCTTCAG
DoDof6-RT-R	CATGGATTAGGCCAAGGAGTAG
DoDof7-RT-F	GGACATTCTCCGTCAGGTACTA
DoDof7-RT-R	GCAGCGAAGCACCAAATAAC
DoDof8-RT-F	GATTACACCCACCGCTACTT
DoDof8-RT-R	GTAGTACGTGGCGGAGAATATC
DoDof9-RT-F	AAGACTGCCGTCGTTATTGG
DoDof9-RT-R	CATAAGGAGCGCTTGGAGTT
DoDof10-RT-F	CATGACACACTTTCAACCACTC
DoDof10-RT-R	GCTGCTCCCAATTTGGTAATC
DoDof11-RT-F	CTTCCACCACCTCCAGTTATT
DoDof11-RT-R	GGAAGAAGACCGTTGAGGAAT
DoDof12-RT-F	AAGAACTCCAAACGGTCTTCC
DoDof12-RT-R	GATCGTCATCTACCGGTGTAAAG
DoDof13-RT-F	GGCGGCTCAAGGAAGAATAA
DoDof13-RT-R	GGCTCAGTAGGCTGTAGATTTG
DoDof14-RT-F	GGATTCTGGCGGTTCGAATA
DoDof14-RT-R	GGCTGTCACGGATCCAATAA
DoDof15-RT-F	GAAGAAGCTCCAGCCTTCAA
DoDof15-RT-R	CCGAAGAAGAAAGTGGTGGT
DoDof16-RT-F	CATGGACCCATCCTCCATTT
DoDof16-RT-R	CACTGCTTGGTGAAGTTGAAAG
DoDof17-RT-F	TTCGGGTTGCAGGAATTAGG
DoDof17-RT-R	GAGGAGCACTTGAGCTAAACA
DoDof18-RT-F	CTATGTCAGCCCTGGAATTGT
DoDof18-RT-R	ACCAGCTCCAAATAGTGATGTC
DoDof19-RT-F	TCCATCCACTTCCCACTACA

DoDof19-RT-R	GGTTGAGCGAGGCAATAGTT
DoDof20-RT-F	GCCATCCAGATCCTCATCAAA
DoDof20-RT-R	GTCGTAGAGGCTTGTATCCATC
DoDof21-RT-F	GACTGCTGGAGGAACAATGA
DoDof21-RT-R	ATCTGGCATAACCACCGTATG
DoDof22-RT-F	CCACCCTTTCTTCTTCTCTTCC
DoDof22-RT-R	AGCTCAGGGAGTTGGTCTAA
DoDof23-RT-F	TGGATCAGGCTACCGTCATA
DoDof23-RT-R	AAGGGCGACAAGGGTTTAG
DoDof24-RT-F	GGCGATATGTGAGGGATTGAG
DoDof24-RT-R	CGGCCAAGCGTAGAGATAAG
DoDof25-RT-F	CTCACCCACCTCTTACTACTCT
DoDof25-RT-R	GGCAATCTACCACCACTGAA
DoDof26-RT-F	TCCTCATCTTCCTCCTCTCTTC
DoDof26-RT-R	GCAGCTGTGTTAGCACCATA
DoDof27-RT-F	CAGTGGAGGTTGCCTTACTT
DoDof27-RT-R	CTCCACCAACACTCCATTCT
DoDof28-RT-F	GAAGACCAGAACTCAACCAGAG
DoDof28-RT-R	AAATAGCGTGGCTGGGAAA
DoACTIN-RT-F	TCCCAAGGCAAACAGAGAAA
DoACTIN-RT-R	GGCCACTAGCATATAGGGAAAG

F, forward; R, reverse

Table S6. Primers used for subcellular localization analysis.

Primer name	Primer sequences (5′→3′)
DoDof15-YFP-F	AGCTCAAGCTTCGAATTCATGGCCGACATCGGCGAAGA
DoDof15-YFP-R	CCGTCGACTGCAGAATTCACACGACTCACTAGCCGCGT
DoDof22-YFP-F	AGCTCAAGCTTCGAATTCATGCTCTCCATCTACTCCTT
DoDof22-YFP-R	CCGTCGACTGCAGAATTCTAGAGAAGCTTCCAAACTCA
DoDof24-YFP-F	AGCTCAAGCTTCGAATTCATGGCGGCTAACATTTACCC
DoDof24-YFP-R	CCGTCGACTGCAGAATTCCAGAAGACGAACCACCGACG

F, forward; R, reverse

Table S7. Primers used for the yeast one-hybrid assay.

Primer name	Primer sequences (5′→3′)
DoDof15-AD-F	GGAGGCCAGTGAATTCATGGCCGACATCGGCGAAGA
DoDof15-AD-R	CACCCGGGTGGAATTCACACGACTCACTAGCCGCGT
DoDof22-AD-F	GGAGGCCAGTGAATTCATGCTCTCCATCTACTCCTT
DoDof22-AD-R	CACCCGGGTGGAATTCTAGAGAAGCTTCCAAACTCA
DoDof24-AD-F	GGAGGCCAGTGAATTCATGGCGGCTAACATTTACCC
DoDof24-AD-R	CACCCGGGTGGAATTCCAGAAGACGAACCACCGACG
DoprobHLH68-HIS-F	ACTATAGGGCGAATTCGTTTGGAAAGCACTGCCACC
DoprobHLH68-HIS-R	AGCTCCCCGGGAATTCGGGTTAAGGCCTGTGTGGTC
DoproPYL9-HIS-F	ACTATAGGGCGAATTCTCATTCAAGTTCTAGTCACA
DoproPYL9-HIS-R	AGCTCCCCGGGAATTCTCTGATTTAAGCTATTATGC

F, forward; R, reverse
